# Cu Promoted the Dynamic Evolution of Ni-Based Catalysts for Polyethylene Terephthalate Plastic Upcycling

**DOI:** 10.1021/acscatal.3c05509

**Published:** 2024-03-26

**Authors:** Hongxing Kang, Dong He, Xingxu Yan, Benjamin Dao, Nicholas B. Williams, Gregory I. Elliott, Daniel Streater, James Nyakuchena, Jier Huang, Xiaoqing Pan, Xiangheng Xiao, Jing Gu

**Affiliations:** † Department of Chemistry and Biochemistry, San Diego State University, 5500 Campanile Drive, San Diego, California 92182, United States; ‡ Department of Physics, Wuhan University, Wuhan, Hubei 430072, China; § Department of Materials Science and Engineering, University of California, Irvine, California 92697, United States; ∥ Department of Physics and Astronomy, University of California, Irvine, Irvine, California 92697, United States; ⊥ Department of Chemistry, California State University, Long Beach, Long Beach, California 90840, United States; # Department of Chemistry, Marquette University, Milwaukee, Wisconsin 53201, United States

**Keywords:** catalyst’s dynamic evolution, direct and indirect oxidation mechanisms, C–C bond cleavage, plastic waste upcycling

## Abstract

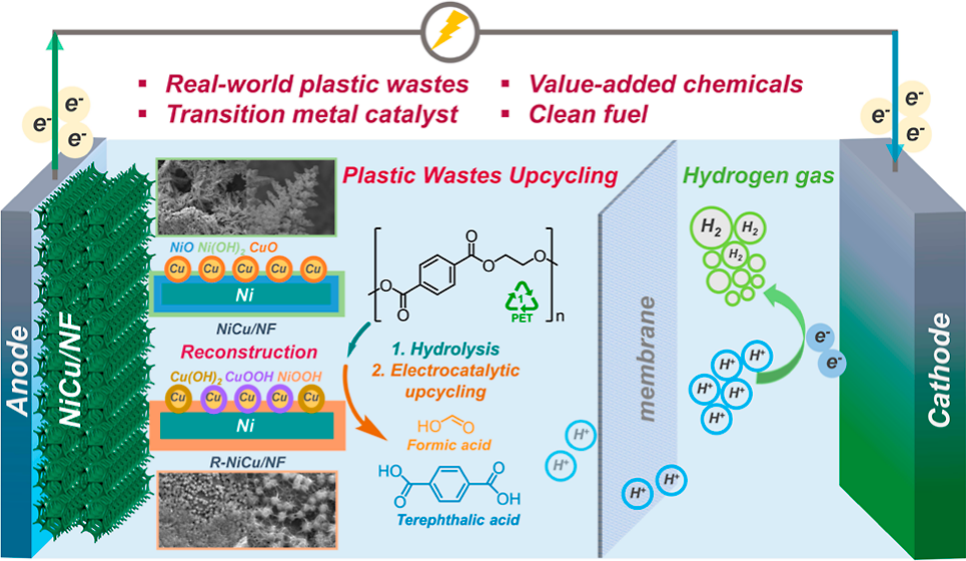

Upcycling plastic wastes into value-added chemicals is a promising approach to put end-of-life plastic wastes back into their ecocycle. As one of the polyesters that is used daily, polyethylene terephthalate (PET) plastic waste is employed here as the model substrate. Herein, a nickel (Ni)-based catalyst was prepared via electrochemically depositing copper (Cu) species on Ni foam (NiCu/NF). The NiCu/NF formed Cu/CuO and Ni/NiO/Ni(OH)_2_ core–shell structures before electrolysis and reconstructed into NiOOH and CuOOH/Cu(OH)_2_ active species during the ethylene glycol (EG) oxidation. After oxidation, the Cu and Ni species evolved into more reduced species. An indirect mechanism was identified as the main EG oxidation (EGOR) mechanism. In EGOR, NiCu_60s_/NF catalyst exhibited an optimal Faradaic efficiency (FE, 95.8%) and yield rate (0.70 mmol cm^–2^ h^–1^) for formate production. Also, over 80% FE of formate was achieved when a commercial PET plastic powder hydrolysate was applied. Furthermore, commercial PET plastic water bottle waste was employed as a substrate for electrocatalytic upcycling, and pure terephthalic acid (TPA) was recovered only after 1 h electrolysis. Lastly, density functional theory (DFT) calculation revealed that the key role of Cu was significantly reducing the Gibbs free-energy barrier (Δ*G*) of EGOR’s rate-determining step (RDS), promoting catalysts’ dynamic evolution, and facilitating the C–C bond cleavage.

## Introduction

Upcycling end-of-life plastics to value-added chemicals and fuels via chemical methods provides an ecofriendly and viable way to address the increasing plastic pollution issues. As one of the most used plastic materials, polyethylene terephthalate (PET) is manufactured almost 70 million tons annually for packaging and textiles.^1^ However, the majority of PET ends its life cycle in landfills,^2^ which is not only harmful to the environment but also represents a massive economic loss.^3,4^ Chemical recycling processes,^5,6^ such as hydrolysis, ammonolysis, methanolysis, and alcoholysis, have been widely applied in industry to degrade PET due to their high efficiencies, low cost, and low energy consumption.^7^ Among them, alkaline hydrolysis is considered one of the most promising methods where PET can be completely depolymerized into terephthalic acid (TPA) and ethylene glycol (EG) monomers. Subsequently, the generated monomers can be purified and manufactured to form new PET bottles or other food-grade polymers.^8,9^ However, no extra economic values can be obtained from this common PET–monomers–PET ecocycle. In view of the current industrial chemical process, there is still great room to integrate PET chemical degradation with upcycling for value-added chemical production.

Recently, electrolysis has emerged as a green and low-energy input approach for PET plastic upcycling.^8−12^ In these works, first, PET undergoes a hydrolysis process to form TPA and EG. Since TPA is quite stable under electrochemical condition, only EG will undergo an ethylene glycol oxidation reaction (EGOR) process, upgrading into value-added products.^13^ However, EGOR usually involves uncontrollable and complicated reaction pathways, leading to the generation of a variety of C_1_ and C_2_ products,^14^ such as formate, glycolate, and oxalate. The poor product selectivity and low efficiency hinder the wide applications of PET upcycling due to the extra downstream energy input requirement for product separation. Therefore, developing selective and efficient electrocatalysts for PET-derived alcohol conversion will be beneficial to cut down the downstream cost.

To design a better catalyst with enhanced selectivity, it is vital to track down the dynamic evolution of catalysts and comprehend their inherent reaction mechanism.^15,16^ Ni- and Cu-based electrocatalysts have been intensively employed for PET waste conversion.^17−19^ For instance, Wang et al.^11^ employed CuO nanowires for PET hydrolysate oxidation, where CuO nanowires can selectively catalyze EG to formate with a Faradaic efficiency (FE) of 86.5%. In a similar work,^8^ a nickel-modified cobalt phosphide (CoNi_0.25_P) was developed to electrochemically convert PET-derived EG into formate and glycolic acid (FE > 80%). More recently, Liu et al.^9^ demonstrated that PET-derived EG can be electro-reformed into glycolic acid over Pd–Ni(OH)_2_ with an excellent selectivity (>90%). Though the abovementioned works laid a great foundation in identifying catalysts for selective PET-derived alcohol oxidation, the dynamic evolution of catalysts, the true active species of these catalysts, and reaction mechanisms are still elusive.^20,21^

In this work, Cu was electrochemically deposited on Ni foam (denoted as NiCu/NF) for EG and PET plastic upcycling. Through extensive ex situ and in situ characterizations, NiOOH and CuOOH/Cu(OH)_2_ were identified as the key active species. Besides, the indirect oxidation mechanism was identified as the main mechanism. Furthermore, density functional theory (DFT) calculations revealed that the Gibbs free energy barrier (Δ*G*) of the EGOR rate-determining step (RDS) was significantly reduced on the NiCu/NF (0.51 eV) compared to that of the pristine Ni foam (NF, 1.41 eV). Meanwhile, the presence of Cu facilitates C–C bond cleavage by lowering Δ*G* of *COOHCH_2_OH to the *CH_2_OH + HCOOH step. Experimentally, the addition of Cu not only significantly promoted the formation of Ni^3+^ active species but also enhanced EGOR kinetics by facilitating interfacial electron transfer. As a result, NiCu_60s_/NF exhibited greatly enhanced FE (95.8%) and yield rate (0.70 mmol cm^–2^ h^–1^) for formate production compared to those of pristine NF (formate, 64.8% and 0.17 mmol cm^–2^ h^–1^). Lastly, commercial PET powder and a PET water bottle were employed. With the PET powder hydrolysate, formate was identified as the main oxidation product (FE > 80%). Selectively converting PET-derived EG to formate as the only conversion product would cut down the downstream cost related to product separation. In addition, pure TPA was regenerated from commercial PET water bottle hydrolysate only after 1 h of electrolysis through a simple acidification and separation process, demonstrating the potential practical application of this method in the real world. By providing a framework for probing the dynamic evolution of catalysts, the true active sites, and the reaction mechanism, this work paves a rational pathway in understanding and designing high-performance electrocatalysts toward more efficient and selective plastic upcycling.

## Results and Discussion

### Structures and Morphologies of NiCu/NF and R-NiCu/NF

**Figure 1:**
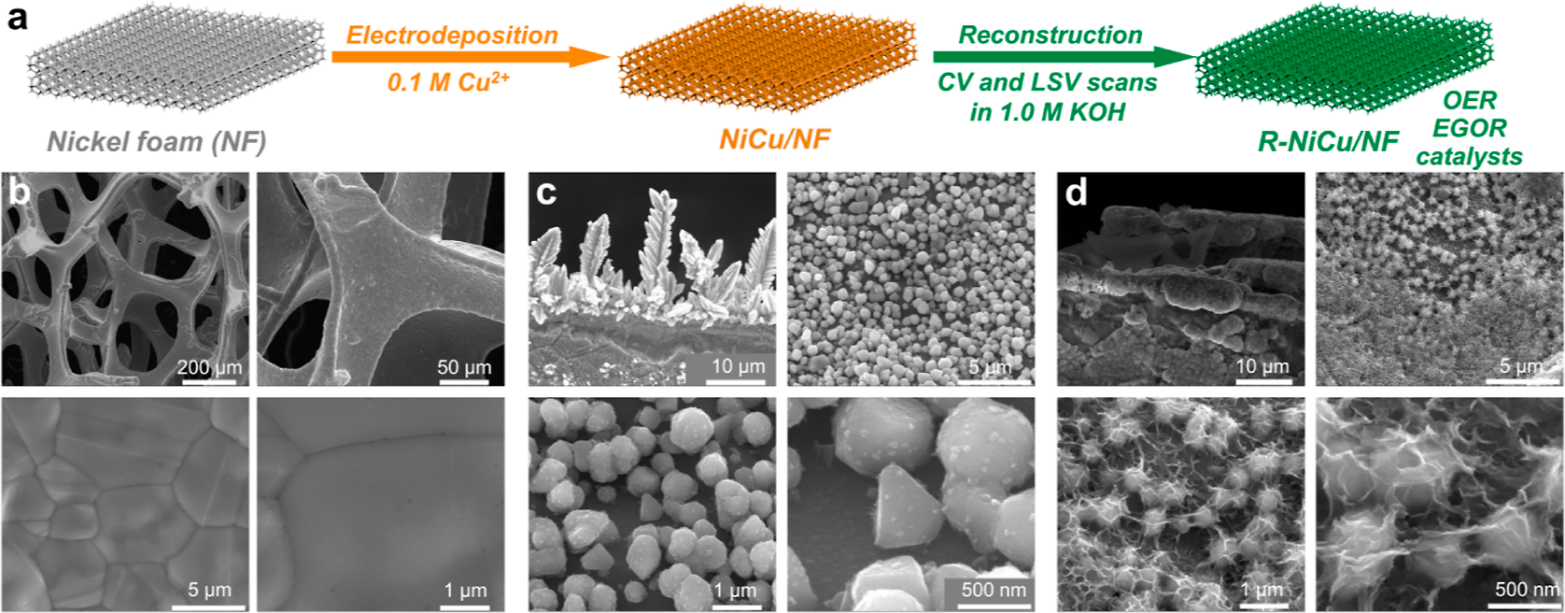
(a) Schematic illustration of the preparation of NiCu/NF and R-NiCu/NF. SEM images of (b) Ni foam, (c) NiCu_60s_/NF, and (d) R-NiCu_60s_/NF; NiCu_60s_/NF was reconstructed in 1 M KOH with 10-cycle CV scans (1.02–1.82 V vs RHE, 100 mV s^–1^) and one LSV scan (1.02–1.82 V vs RHE, 10 mV s^–1^).

The self-supported NiCu/NF electrodes were prepared by cathodic electrodeposition of Cu species on the NF surface (Figures 1a and S1). The optimal deposition time was identified as 60 s. Thus, the structural and morphological characterizations were based on NiCu_60s_/NF if no specific notifications were made. Further, an active EGOR catalyst was formed after a reconstruction process (R-NiCu/NF) through cyclic voltammetry (CV) and linear sweep voltammetry (LSV) scans (see Supporting Information for details). Herein, the oxygen evolution reaction (OER) was employed as a control reaction for EGOR to understand the evolution processes of the catalysts.

**Figure 2:**
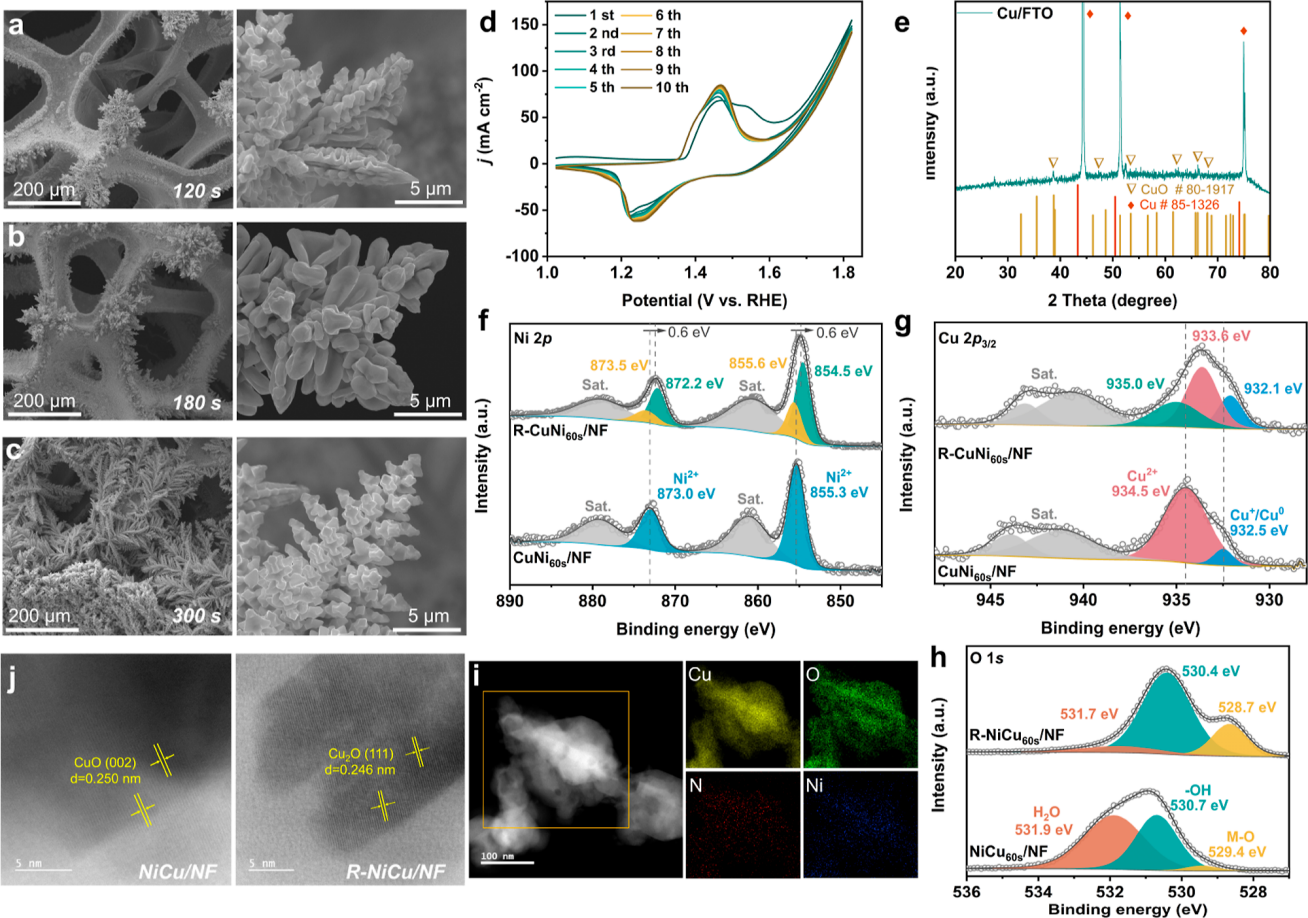
SEM images of NiCu/NF with different electrodeposition times. (a) 120, (b) 180, and (c) 300 s (d) CV scans of NiCu_60s_/NF in the reconstruction process. (e) XRD patterns of Cu species on fluorine-doped tin oxide (FTO) to avoid the interferences of NF. XPS spectra of (f) Ni 2p and (g) Cu 2p_3/2_; the Cu 2p_3/2_ peak can be deconvoluted into two peaks, where the peak at 932.5 eV can be ascribed to the formation of Cu^0^ or diamagnetic Cu_2_O (Cu^+^). Unfortunately, these two species are indistinguishable in the Cu 2p_3/2_ spectra. (h) O 1s. (i) High-angle annular dark-field STEM image and the corresponding EDS elemental mapping results of Cu, O, N, and Ni for Cu species on the NiCu/NF sample. (j) Bright-field STEM images of Cu species of NiCu/NF and R-NiCu/NF. Herein, the electrodeposition time was set to 30 min for the STEM measurements.

The structure and morphology of the as-prepared electrodes were characterized by scanning electron microscopy (SEM). Commercial NF (Figures 1b and S2a) and Cu foam (CF) (Figure S2b) are composed of frames and pores of hundreds of micrometers. As shown in Figures 1c and S3a, the electrodeposited NiCu_60s_/NF displays fine dendritic frames and nanoparticles on the NF substrate. After reconstruction, more hierarchical wrinkles were observed on the nanoparticle surface of R-NiCu_60s_/NF (Figures 1d and S3b). In addition, the dendrite length was obviously elongated with an increased deposition time (Figures 2a–c and S4). The dendritic growth is ascribed to the local depletion of the Cu ion concentration.^22^ More specifically, when a large cathodic bias is applied to the NF, the reduction speed of Cu ions will be much faster than their diffusion speed, resulting in the dendritic growth of the Cu species.^22^

The reconstruction processes of NF, Cu foam (CF), and NiCu/NF electrodes were confirmed by the dynamic change of CV cycles. For instance, the first CV curve of NiCu/NF exhibits a clear oxidation of Ni^2+^/Ni^3+^ at ∼1.45 V (vs RHE), followed by a sharp increased anodic current (Figures 2d and S5a) of OER (>1.6 V vs RHE).^23^ The successive 1st to 10th cycles display a gradually decreased OER current density and a slightly shifted Ni^2+^/Ni^3+^ peak position. However, after 10 CV cycles, the aforementioned changes become negligible. This result indicates that the NiCu/NF electrocatalyst undergoes a rapid structural reconstruction during the CV cycles. In addition, similar OER current density changes and shifts of redox peak positions were observed on NF and CF (Figure S5b,c), indicating that these electrodes also undergo reconstructions.

### Reconstruction and Core–Shell Structure of NiCu_60s_/NF

X-ray diffraction (XRD) was further performed to investigate the chemical states of the deposited Cu species (Figures 2e and S6). The XRD patterns of Cu species show characteristic peaks of Cu (PDF 85-1326) and CuO (PDF 80-1917), indicating the formation of Cu and CuO.

The chemical environment and electronic properties of NiCu/NF before and after the reconstruction processes were further investigated by X-ray photoelectron spectroscopy (XPS). Before reconstruction, the deconvoluted Ni 2p spectrum of NiCu_60s_/NF displays two main peaks accompanied by two satellite peaks (Figure 2f and Table S1). The peaks at 855.3 eV (Ni 2p_3/2_) and 873.0 eV (Ni 2p_1/2_) are ascribed to the existence of Ni(OH)_2_.^24^ This result is supported by Fourier transform infrared spectroscopy (FTIR), where the –OH stretching and deformation vibrations at 3221 and 1362 cm^–1^ indicate the presence of metal hydroxide species (Figure S7). It is noticeable that metallic Ni (Ni^0^) was not detected by XPS, which can be explained by the existence of NiO, Ni(OH)_2_, and the CuO overlayer. After reconstruction (in R-NiCu_60s_/NF), besides the original Ni(OH)_2_ species, the Ni 2p_3/2_ peak (854.5 eV) revealed the coexistence of NiO.^24,25^ The presence of NiO and Ni(OH)_2_ on the surface of R-NiCu_60s_/NF can stem from the following reasons: first, NiOOH generated under the oxidation condition can be rapidly consumed and reduced back to Ni(OH)_2_ coupled with OER. As well, the reaction between Ni^0^ and OH^–^ can produce Ni(OH)_2_. Lastly, the dehydration of NiOOH when exposed to air can result in the formation of NiO.^26^ In addition, after the reconstruction, the Ni 2p spectrum (Figure 2f) shows a negative shift of binding energy (0.6 eV), indicating a decreased electron density of the surface Ni species.

In terms of Cu species, before reconstruction, the Cu 2p_3/2_ peak (Figure 2g) of NiCu_60s_/NF can be deconvoluted into two peaks, where the peak at 932.5 eV is ascribed to the presence of either metallic Cu (Cu^0^) or diamagnetic cuprous oxide (Cu^+^, Cu_2_O).^27^ Meanwhile, cupric oxide (CuO) with a binding energy of 934.5 eV can be identified.^28,29^ The presence of CuO can be further supported by the presence of a strong shakeup satellite peak of Cu 2p_3/2_ (shown as Sat., located in the range of BE = 938.0–947.6 eV) due to its paramagnetic properties.^27,30^ One interesting aspect is that Cu deposition was conducted under a pH of 4.15 at −0.956 V (vs Ag/AgCl) in a 0.1 M Cu(NO_3_)_2_ solution. Under this condition, Cu^2+^ ions should be reduced to metallic Cu^0^ according to the Pourbaix diagram (Figure S8a).^31^ However, ex situ XPS results indicate that the surface layer is mainly composed of CuO. Thus, it is reasonable to hypothesize that the surface Cu^0^ can be spontaneously oxidized to CuO when exposed to air. This result suggests the formation of a core–shell structure where Cu^0^ core might lie beneath the CuO shell on the NiCu_60s_/NF surface.^32^ To prove this hypothesis, NiCu_60s_/NF and NiCu_180s_/NF samples were dried under 60 °C for 3 and 45 h prior to the XPS measurements (see XPS analysis section in Supporting Information for more details). Briefly, the 45 h air-exposed sample contains more CuO than the 3 h sample (Figure S9). This result confirms that air oxidation is the major reason for the formation of surface CuO. Additionally, in an Ar^+^-sputtering process, from 0 to 5 min, the percentage of CuO species was reduced from 34.43 to 15.32%, while the percentage of Cu^+^/Cu^0^ increased from 3.64 to 46.56% (Figures S11 and S12), confirming the existence of the Cu^0^ core. After reconstruction, Cu 2p_3/2_ of R-NiCu_60s_/NF exhibits three prominent peaks and two satellite peaks. Besides the peaks of Cu^+^/Cu^0^ (932.1 eV) and CuO (933.6 eV), the peak at 935.0 eV is assigned to the presence of Cu(OH)_2_. The presence of Cu(OH)_2_ might result from reacting Cu^+^/Cu^0^ with OH^–^ during the reconstruction process. In addition, the FTIR spectrum further confirms the existence of Cu(OH)_2_ in the presence of –OH stretching and deformation vibrations (Figure S7).

O 1s spectrum corresponds well with the aforementioned metal species assignments (Figure 2h). Herein, the O 1s XPS spectrum of NiCu_60s_/NF was deconvoluted into three peaks, 529.4, 530.7, and 531.9 eV, corresponding to the presence of M–O (CuO), hydroxide (M–OH), and adsorbed water,^33,34^ respectively (Table S1). After reconstruction, the O 1s spectrum of R-NiCu_60s_/NF shows the coexistence of three O species: the metal oxide (M–O, 528.7 eV), hydroxide (−OH, 530.4 eV), and adsorbed water (H_2_O, 531.7 eV), respectively. Compared to the NiCu_60s_/NF, a higher percent of M–O species at 528.7 eV was present (Table S2). The increased number of M–O species can be ascribed to the transformation of metallic species into their corresponding metal oxides (Cu to CuO and Ni to NiO) and NiOOH to NiO after reconstruction.

Further, the oxidation state and local structure changes of the catalyst were studied by X-ray absorption spectroscopy (XAS). After reconstruction (in R-NiCu_60s_/NF), the Ni K-edge shifts to a higher energy compared to NiCu_60s_/NF in the X-ray absorption near-edge structure (XANES) spectrum, indicating that the average oxidation state of Ni was increased (Figure S14a).^23,35^ In addition, in the Fourier transform extended fine structure spectroscopy (FT-XAFS, Figure S14b), the characteristic features of NiCu_60s_/NF were located at 1.47 and 2.49 Å, corresponding to Ni–O and Ni–Ni correlations, respectively.^36,37^ The radial distance of Ni–O was down-shifted to 1.26 Å, while Ni–Ni remains the same in R-NiCu_60s_/NF, further confirming the elevated oxidation states of Ni species after reconstruction.^38,39^

### EDS and STEM Analyses

Moreover, scanning transmission electron microscopy (STEM) and energy-dispersive X-ray spectroscopy (EDS) were carried out to identify the composition and distribution of the Cu species. Herein, electrodeposition time was set to 30 min due to the difficulty in observing the Cu species with a shorter deposition time (see Supporting Information for details). As shown in Figures 2i and S15, after electrodeposition, Cu and O were observed with a Cu/O ratio of ∼6 in NiCu_30min_/NF. However, after the reconstruction process, the Cu/O ratio was decreased to ∼3 in the R-NiCu_30min_/NF, indicating the formation of more oxidized species (Figure S16). This result further suggests the existence of the Cu^0^/CuO core–shell structure, where the Cu^0^ core can be gradually oxidized in the reconstruction process. Furthermore, the Cu species before reconstruction show a clear lattice fringe with 0.250 nm *d*-spacing, corresponding to the (002) plane of CuO,^40,41^ which is in good agreement with the XRD and XPS results. After reconstruction, the interplanar spacing changed to 0.246 nm, which is ascribed to the (111) plane of Cu_2_O.^42^ The formation of Cu_2_O might have originated from the in situ-formed Cu(OH)_2_ and CuOOH (see Cu active species identification section in Supporting Information) that undergo dehydration and reduction during the reconstruction process.

### EGOR Performance

**Figure 3:**
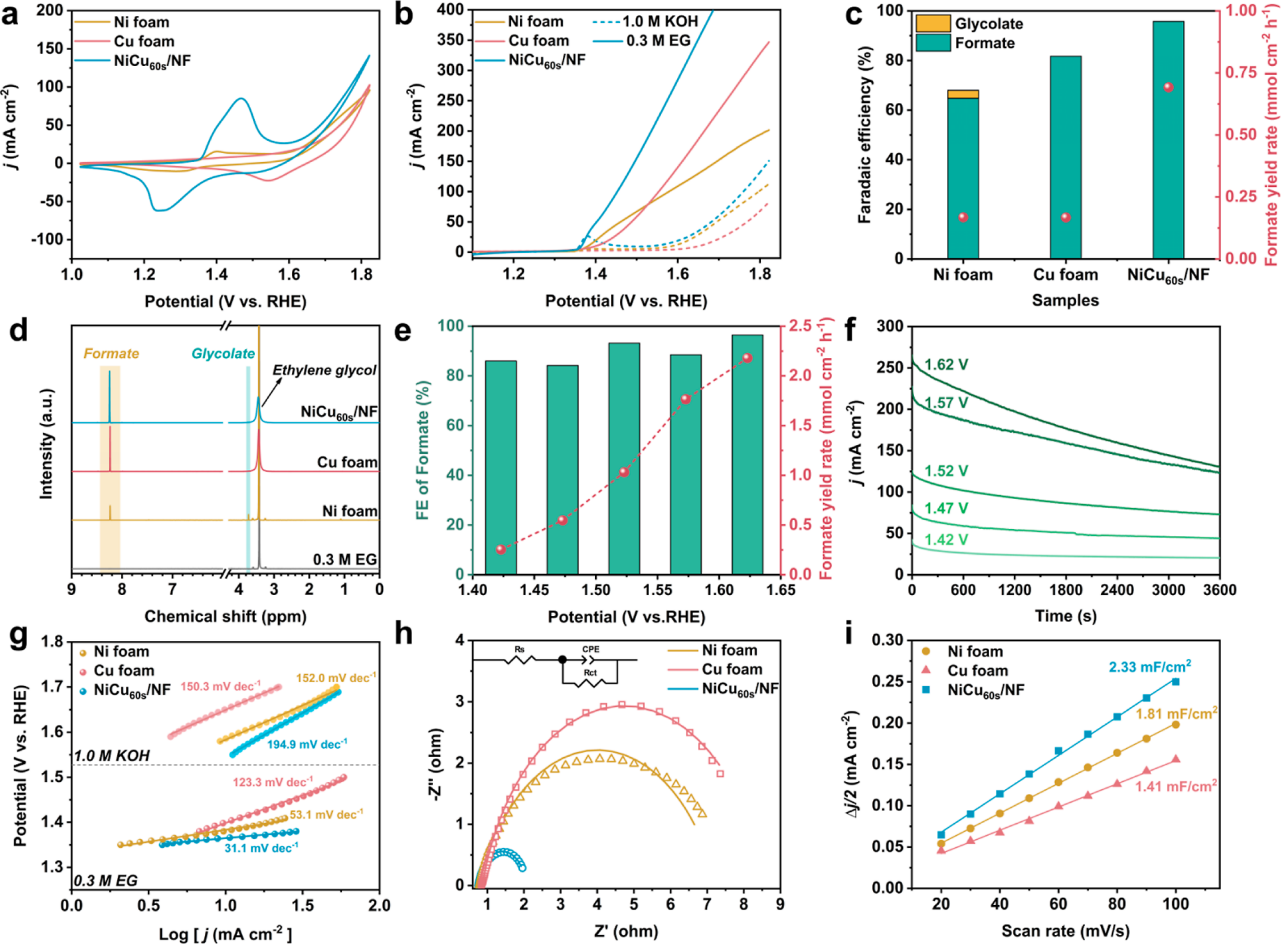
Ni foam, Cu foam, and NiCu_60s_/NF electrocatalytic performance in 1.0 M KOH. (a) CV curves and (b) LSV curves (without IR correction) for the OER and EGOR (with the addition of 0.3 M EG) in 1.0 M KOH. (c) FEs and yield rates of formate of different electrodes at 1.47 V vs RHE after 2 h electrolysis. (d) ^1^H NMR spectra of the electrolytes before and after electrolysis. (e) FEs and yield rates of formate of the NiCu_60s_/NF electrode for EGOR over 1 h electrolysis at different applied potentials. (f) Chronoamperometry (*i*–*t*) curves from 1.42 to 1.62 V. (g) Tafel slopes in 1.0 M KOH with and without the addition of 0.3 M EG. (h) Electrochemical impedance spectra and the EIS fitting parameters presented in Table S5. (i) Calculated double-layer capacitance of NF, CF, and NiCu_60s_/NF, respectively.

The electrocatalytic performance of NiCu/NF for EGOR was carried out in a three-electrode H-cell equipped with a Nafion membrane (Figure S17a). Herein, if not specifically mentioned, all electrochemical measurements are referenced with a reversible hydrogen electrode (RHE) in 1.0 M KOH. First, the redox chemistry of NiCu/NF was investigated by CV (Figure 3a). Interestingly, we noticed an enhanced anodic peak of Ni^2+^/Ni^3+^ (at ∼1.45 V) in NiCu_60s_/NF compared to that of NF, suggesting that the incorporation of Cu facilitates the formation of Ni^3+^. In addition, the addition of Cu also greatly increases the catalyst selectivity indicated by a greater potential difference (Δ*E*) between EGOR and OER to achieve 100 mA cm^–2^ (Figure 3b). Further, the electrodeposition time was optimized based on the EGOR performance (Figure S17b–e). NiCu/NF electrodes with varied electrodeposition times (15–300 s) displayed a wide range of Δ*E* from 255 to 307 mV. Among them, the optimal electrodeposition time was identified to be 60 s with an optimal Δ*E* (307 mV), formate selectivity (FE, 95.8%), and yield rate (0.70 mmol cm^–2^ h^–1^). Herein, formate was identified as the sole product, quantified by ^1^H and ^13^C NMR (Figures S18–S20).

In comparison, a small amount of C_2_ product, glycolate (FE, 3.22%), together with the C_1_ product, formate (FE, 64.8%), was detected when a NF electrode was employed. In addition, formate with lower productivity (FE, 81.7%, and yield rate, 0.17 mmol cm^–2^ h^–1^) was noticed on a CF electrode (Figure 3c,d). This result indicates that the incorporation of Cu species not only promotes the formation of active species but also facilitates the breakage of the C–C bond. The FE of formate with the NiCu_60s_/NF electrode is maintained constantly higher than 85% from 1.42 to 1.62 V versus RHE (Figures 3e and S21), and the productivity of formate is enhanced compared to those of the bare NF and CF (Figure S22). Furthermore, chronoamperometry (*i*–*t*) curves at different applied potentials display a rapid current density depletion resulting from the fast consumption of EG, indicating the superior efficiency of NiCu_60s_/NF (Figure 3f). Also, the improved EGOR kinetics of NiCu_60s_/NF was verified by a much-lowered Tafel slope of 31.1 mV dec^–1^ than those of NF (53.1 mV dec^–1^) and CF (123.3 mV dec^–1^) (Figure 3g). Electrochemical impedance spectroscopy (EIS) measurements were further carried out to investigate the overall kinetics. Here, the Nyquist plots (Figure 3h) showed a much smaller semicircle diameter of NiCu_60s_/NF than those of NF and CF, suggesting that the addition of Cu significantly increases the interfacial charge-transfer kinetics. In addition, calculating the double-layer capacitance (*C*_dl_) sheds light on the changes in the electrochemical active surface area (ECSA) (Figures 3i and S23). *C*_dl_ of NiCu_60s_/NF was calculated to be 2.33 mF/cm^2^, which is 1.28 and 1.65 times better than those of NF and CF, respectively. This result indicates that NiCu_60s_/NF has an increased active surface area compared to those of the bare NF and CF.

Furthermore, the stability of NiCu_60s_/NF was evaluated under short-term (2 h) and long-term (24 h) electrolysis. As shown in Figure S24a, after 1 h of electrolysis, the current density was recovered by refreshing the electrolyte. In addition, a slightly decreased FE and EG conversion was observed in the second electrolysis cycle (Figure S24b), suggesting that the gradual reduction of current density was mainly caused by the consumption of EG rather than the deconstruction of the catalyst. Further, the surface structures of NiCu_60s_/NF were analyzed and compared after the OER and EGOR. As shown in the XPS spectra (Figure S25), CuO (933.7 eV) and Ni(OH)_2_ (855.2 eV) were observed after the OER for 2 h. Interestingly, more reduced forms Cu^+^/Cu^0^ (931.5 eV) and Ni^0^ (852.0 eV) were identified after EGOR. The presence of low-oxidation metal species after EGOR compared to OER is attributed to the fact that EG is a stronger reduction reagent than water (OH^–^),^43^ which can reduce active Ni^3+^ and/or Cu^2+^/Cu^3+^ species (see In Situ Raman Section in the Supporting Information) to their lower oxidation states via an indirect oxidation mechanism, which will be further elaborated in the Reaction Mechanism Section (in the Supporting Information). The presence of more reduced Ni species after EGOR was further confirmed by XAS (Figure S14). Specifically, after EGOR, the observed Ni K-edge shifted to a lower energy compared to the unreacted NiCu_60s_/NF (Figure S14a), implying the formation of more reduced Ni species. Meanwhile, the weaker peak intensity of Ni–O in the post-EGOR NiCu_60s_/NF (Figure S14b) also indicates the reduction of the oxidation state of the Ni species. Furthermore, a long-term stability test (24 h) shows a similar but more significant current density depletion as compared to the short-term stability test (2 h) (Figure S26). As well, the XPS spectra of NiCu_60s_/NF after long-term electrolysis (Figure S27) show the presence of similar Cu and Ni species as that of 2 h electrolysis (Figure S25).

### True Active Species of Ni and Differences between EGOR and OER

**Figure 4:**
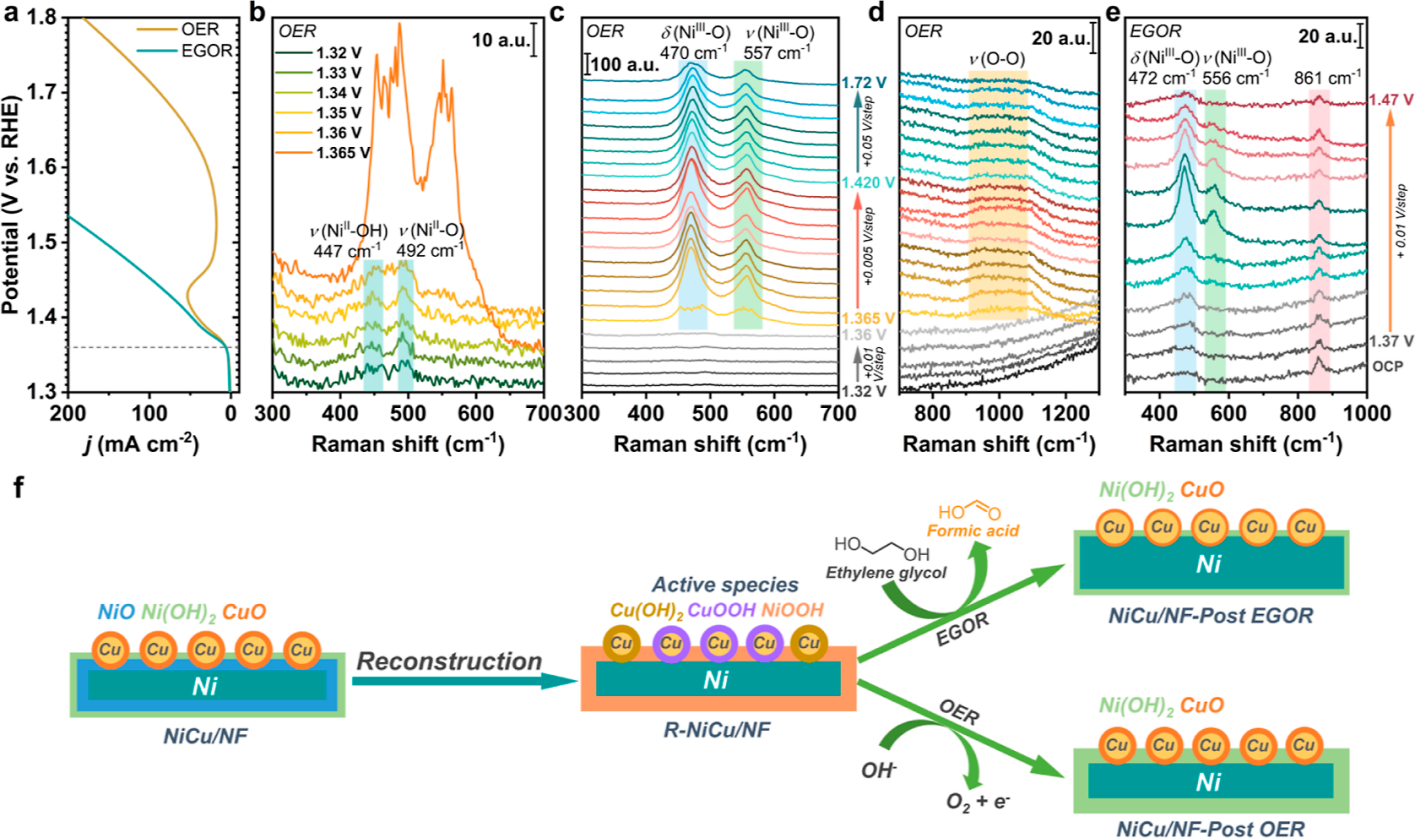
In situ Raman spectroscopy carried out in a homemade electrochemical cell filled with a 1.0 M KOH electrolyte (Figure S28). (a) LSV curves of NiCu_60s_/NF under the conditions of OER (1.0 M KOH) and EGOR (1.0 M KOH with 0.3 M EG). (b–d) In situ Raman spectra of NiCu_60s_/NF for OER and (e) EGOR. Herein, the Raman peak at 863 cm^–1^ is ascribed to the C–C stretching vibration of EG. (f) Schematic illustration of Ni and Cu species changes during the reconstruction and after EGOR and OER.

A higher bias than the Ni^2+^/Ni^3+^ redox potential (∼1.365 V vs RHE, Figure 4a) was applied for EGOR, suggesting Ni^3+^ species should be in situ-generated in the conversion process. However, Ni^3+^ species was not directly detected by ex situ techniques, such as XPS and STEM. To track down the active species during the OER and EGOR, in situ Raman spectroscopy was employed. In Figure 4b, when the applied potentials were smaller than 1.365 V, two Raman peaks at 447 and 492 cm^–1^, corresponding to A_1g_ stretching modes of Ni^II^–OH and Ni^II^–O,^26,44,45^ respectively, were identified. Herein, the Ni–O stretching mode has been ascribed to a potential-assisted dehydration process happening on the transition of Ni(OH)_2_ to NiO.^26^ When the applied potentials were higher than 1.365 V, two new peaks appeared at 470 and 557 cm^–1^ (Figures 4b,c), which are correlated to the E_g_ bending and the A_1g_ stretching vibrations, respectively, of Ni^III^–O in NiOOH.^26,45,46^ Furthermore, Figure 4d shows the development of a broad peak of “active oxygen”^26^ in the region ∼900–1150 cm^–1^ in the bias range of 1.365–1.72 V, confirming the formation of NiOOH species. Moreover, in comparison with the Raman peak intensity of NiOOH, the Raman peak intensity of Ni(OH)_2_ is much weaker. This phenomenon can be explained by the previous work from Yeo and Bell,^46^ where the cross section of Ni(OH)_2_ suffers from a low Raman scattering. In contrast, NiOOH has a strong Raman intensity due to the resonance enhancing effect.^46^

In the presence of 0.3 M EG (Figure 4e), when the applied potentials were greater than 1.41 V, clear E_g_ bending (∼472 cm^–1^) and the A_1g_ stretching (∼556 cm^–1^) vibrations, corresponding to the existence of NiOOH, were observed, and these two peaks reached their maximum intensities at 1.43 V, suggesting NiOOH is the active Ni species for EGOR. In addition, it is noticed that the E_g_ and A_1g_ peak intensities of Ni^III^–O under the EGOR condition are much lower than those under OER, probably resulting from the rapid consumption of NiOOH in the presence of EG. This result strongly supports the previous observation of structural change between EGOR and OER, where lower oxidation states Ni and Cu species were generated after EGOR compared to OER. Combining the ex situ and in situ results, it is reasonable to conclude that the in situ-generated NiOOH is the active Ni species, which is rapidly consumed and reduced back to its lower oxidation-state species in both EGOR and OER.

### True Active Species of Cu

To understand the role of the Cu species, CV and in situ Raman spectroscopy were conducted. As shown in previous CV (Figure 3a) and Raman spectroscopy (Figure 4b–e), the characteristic Cu species’ peaks are absent due to its low deposition amount compared to NF. To exclude the interference of the NF substrate, CF, carbon fiber paper (CFP), and fluorine-doped tin oxide (FTO) were employed as substrates, respectively (see Supporting Information).

From the CV studies, it is observed that CF, Cu/CFP, and Cu/FTO all displayed a similar redox chemistry. Three anodic peaks and three cathodic peaks were identified (Figure S29a–d). The peak a at ∼0.62 V versus RHE is attributed to the Cu^0^ to Cu^1+^ transition, while the peak b (0.82 to 1.02 V vs RHE) is assigned to two possible oxidation processes: Cu^0^ to Cu^2+^ and Cu^1+^ to Cu^2+^. Following that, oxidation peak c (∼1.52 V vs RHE) is associated with the conversion of Cu^2+^ to Cu^3+^. Beyond the potential of peak c, the presence of Cu^3+^ might induce the formation of CuOOH.^47,48^ The corresponding anodic and cathodic reactions are displayed in Figure S29b. CV studies indicate the coexistence of CuOOH and Cu(OH)_2_, which may synergistically serve as active species.

Furthermore, in situ Raman spectroscopy provides more direct evidence of the true Cu active species. Under OER condition (Figure S30a), with applied potentials between 1.32 and 1.62 V, a small peak at ∼483 cm^–1^ was observed, ascribed to the formation of Cu(OH)_2_.^49,50^ When the applied potential was >1.62 V, a new peak evolved at 570 cm^–1^, corresponding to the formation of CuOOH species.^51^ This result was supported by the DFT calculation (Figure S30c), where CuOOH vibration peaks at ∼500 and ∼550 cm^–1^ were noticed. Under the EGOR condition (Figure S30b), the characteristic peaks of Cu(OH)_2_ (at ∼491 cm^–1^) and CuOOH (at ∼565 cm^–1^) were detected when the applied potential was >1.50 V vs RHE (Cu^2+^/Cu^3+^ redox potential). These results indicate that the in situ-formed Cu(OH)_2_ and CuOOH simultaneously serve as the active species for EGOR and OER (see Supporting Information for more details).

Combining the in situ and ex situ results, Cu^0^ was deposited on NF to form NiCu_60s_/NF. The Ni species exist as Ni^0^, NiO, and Ni(OH)_2_, while the Cu species form a Cu^0^/CuO core–shell structure. After reconstruction, Cu(OH)_2_/CuOOH and NiOOH were identified as the active sites. The active species were further converted to the corresponding lower oxidation state metal species after EGOR and the OER (Figure 4f).

### Indirect/Direct Reaction Mechanisms

Indirect and direct oxidation processes are two dominant mechanisms for electrochemical alcohol oxidation reactions.^52^ For the indirect oxidation mechanism of NiOOH,^53−55^ in the first step, Ni(OH)_2_ will be oxidized to NiOOH, which serves as a chemical oxidant and abstracts an α-hydrogen atom from the adsorbed alcohol, which is the rate-determining step (RDS) of the electrochemical oxidation process. In this step, NiOOH was converted back to Ni(OH)_2_. The main function of the applied bias is to regenerate NiOOH as an oxidant. On the contrary, in a direct oxidation process, potential-dependent alcohol oxidation^56−58^ was commonly observed. According to the pioneer work of Choi et al., the direct oxidation mechanism is usually coupled with the formation of Ni^4+^, together with a hydride transfer as a seeming dehydrogenation mechanism.^55,59^ As aforementioned, the characteristic NiOOH peaks at the same applied potential are much smaller under EGOR than those under the OER. The lower NiOOH peak intensity under EGOR conditions can be explained by the rapid consumption of NiOOH via chemically oxidizing EG, indicating that the mechanism of EGOR over NiCu/NF might be an indirect process (Schemes S1 and S2).

**Figure 5:**
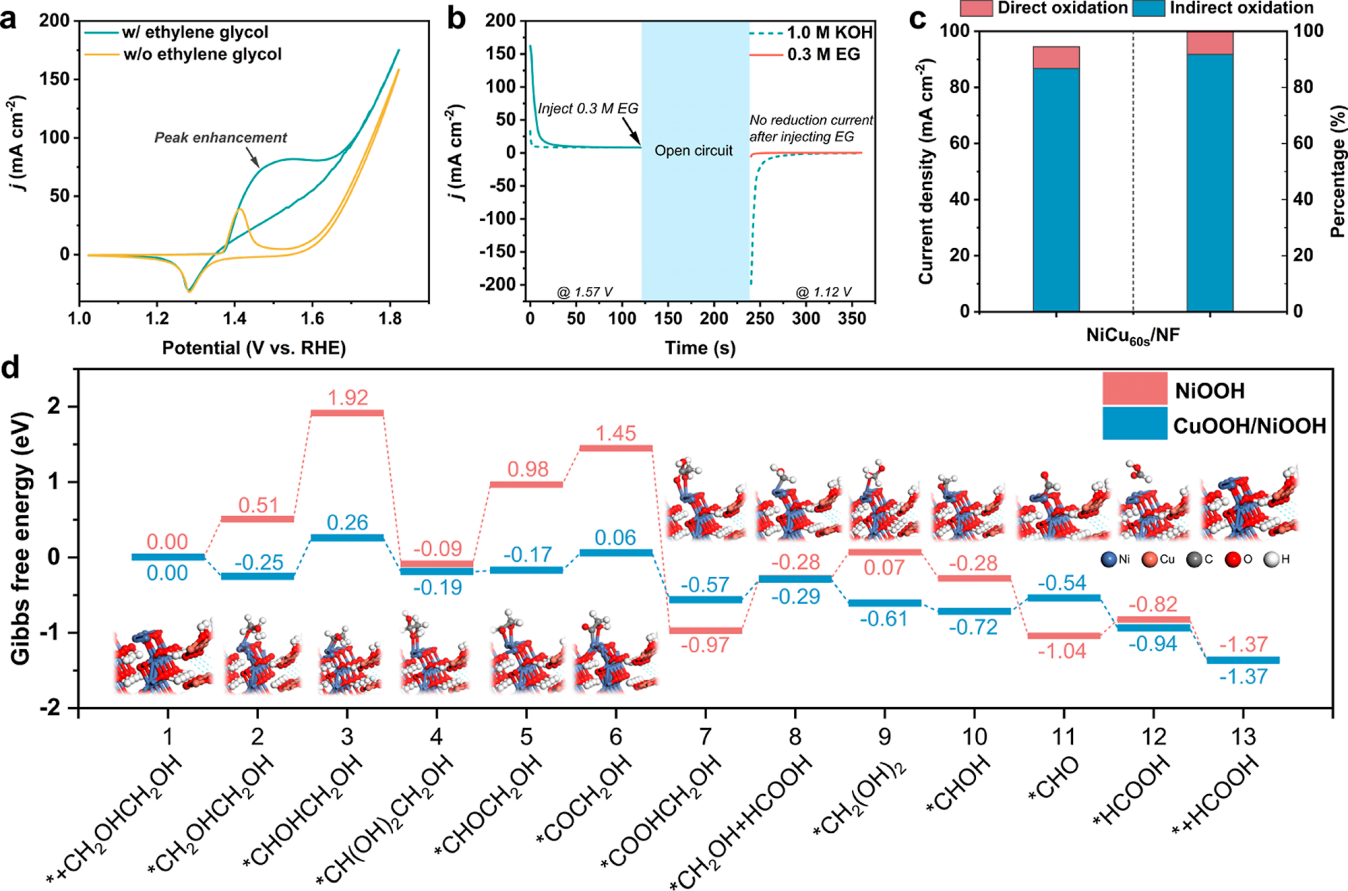
(a) CV curves using a NiCu_60s_/NF electrode in 1.0 M KOH with and without 0.3 M EG. (b) Multipotential step curves of NiCu_60s_/NF. (c) Contribution of the indirect and direct oxidation reactions for EGOR at 1.52 V vs RHE. (d) Gibbs free-energy diagram of EGOR on NiOOH and NiOOH/CuOOH. The numbers are the Gibbs free energies for each state, with units in eV. Since the lattice mismatch of NiOOH/CuOOH and Cu(OH)_2_, Cu(OH)_2_ is not included in the DFT calculation.

To confirm this hypothesis, CV scans were performed with and without the presence of EG. Without EG, the oxidation and reduction transitions of Ni^2+^/Ni^3+^ show a similar intensity (Figure 5a). With EG, the Ni^2+^ to Ni^3+^ anodic peak enhancement was observed, indicating that NiOOH was chemically converted back to Ni(OH)_2_ under the EGOR condition. To further confirm the indirect oxidation mechanism, *i*–*t* tests were conducted (Figure 5b). In 1.0 M KOH, when an anodic potential of 1.57 V was applied on NiCu_60s_/NF for 120 s, an oxidation current was observed, which is ascribed to the oxidation of Ni(OH)_2_ to NiOOH. After the withdrawal of the applied potential, 0.3 M EG was injected into the electrolyte during the open-circuit condition. After that, a 1.12 V potential was applied. It is expected that if NiOOH is chemically consumed, no reduction current should be observed. In contrast, if NiOOH is not consumed, NiOOH to Ni(OH)_2_ reduction should be generated. After EG injection, no reduction current was observed, suggesting that in situ-formed NiOOH was chemically consumed by oxidizing EG. Furthermore, the product of EGOR chemical oxidation was quantified (see Supporting Information for details). Formate was identified as the product (Figure S31a), and the concentration of formic acid was maintained at ∼1.4 μmol/mL when the chemical oxidation reaction time extended from 10 to 300 min, indicating the chemical oxidation reaction finished at around 10 min (Figure S31b).

To quantitatively deconvolute the contribution of indirect and direct oxidation of EG over NiCu_60s_/NF, a three-step electrochemical procedure was applied (Figure S32) (see reaction mechanism section in Supporting Information). Briefly, in this three-step procedure, the plot of 1/remained charge (1/*C*) versus indirect oxidation time (*t*) displays a linear relationship, which allows the charge disappearance rate from the electrode to be calculated via a pseudo-second-order rate law, . Herein, the instantaneous rate of charge disappearance from the electrode is equal to the indirect oxidation current, . Therefore, the direct oxidation current can be calculated from the total current subtracting the indirect oxidation current: *I*_direct_ = *I*_total_ – *I*_indirect_.

As shown in Figure 5c, indirect oxidation of EG exhibits a 92.1% contribution to the total current density, while direct oxidation’s contribution is 7.9% (at 1.52 V vs RHE). Although only one potential was used to decouple the indirect and direction oxidation reactions, considering the direct oxidation usually occurs at potentials more positive than the potentials that enable indirect oxidation, we can conclude that the indirect oxidation pathway is the major pathway for EGOR over NiCu/NF.

### DFT Calculation

Based on the literature, two major EGOR reaction pathways are proposed (Figure S35a). In reaction Pathway 1, EG will be oxidized via two subsequent two-electron oxidation reactions to glycoladehyde and glycolic acid. Further, glycolic acid can undergo C−C bond cleavage and another two-electron oxidation to form formic acid. In Pathway 2, glycolaldehyde will be oxidized to glyoxal, followed by formic acid through two subsequent two-electron oxidation reactions. Here, glycolaldehyde could be oxidized to either glycolate or glyoxal. To determine which pathway is the dominant pathway, glycolic acid and glyoxal were employed as starting materials for oxidation. Herein, glycolic acid oxidation is thermodynamically and kinetically easier than glyoxal, as shown in Figure S35b, suggesting that Pathway 1 (EG–glycolic acid–formic acid) is the dominant pathway (Figure S35a). Therefore, a DFT calculation was further conducted on Pathway 1 to shed light on the role of Cu. Herein, NiOOH was used as the active species for NF, while the CuOOH/NiOOH composite was applied as the active species for NiCu/NF. The optimized structures for NiOOH/CuOOH and NiOOH are displayed in Figures S36 and S37, respectively. From the theoretical prediction, the EG adsorption process (steps 1–2) is endothermal on NiOOH, while it is exothermal on CuOOH/NiOOH (Figure 5d). This result indicates that the addition of Cu facilitates the adsorption of EG. Further, in the EG dehydrogenation process (steps 2–3), which is the RDS for both catalysts, CuOOH/NiOOH shows a much lower Gibbs free-energy barrier (Δ*G*, 0.51 eV) compared to NiOOH (1.41 eV), which is one of the main reasons for the superior EGOR performance on NiCu/NF. Furthermore, CuOOH/NiOOH exhibits a lower C–C bond cleavage Δ*G* for the generation of first formic acid (steps 7–8, *COOHCH_2_OH to *CH_2_OH + HCOOH), indicating the presence of Cu that facilitates C–C cleavage. This result correlates well with the electrochemical conversion results, where glycolate was identified as another product besides formate when NF was used, while only formate was identified as the sole product on the NiCu/NF electrode.

### Electrocatalytic PET Upcycling

**Figure 6:**
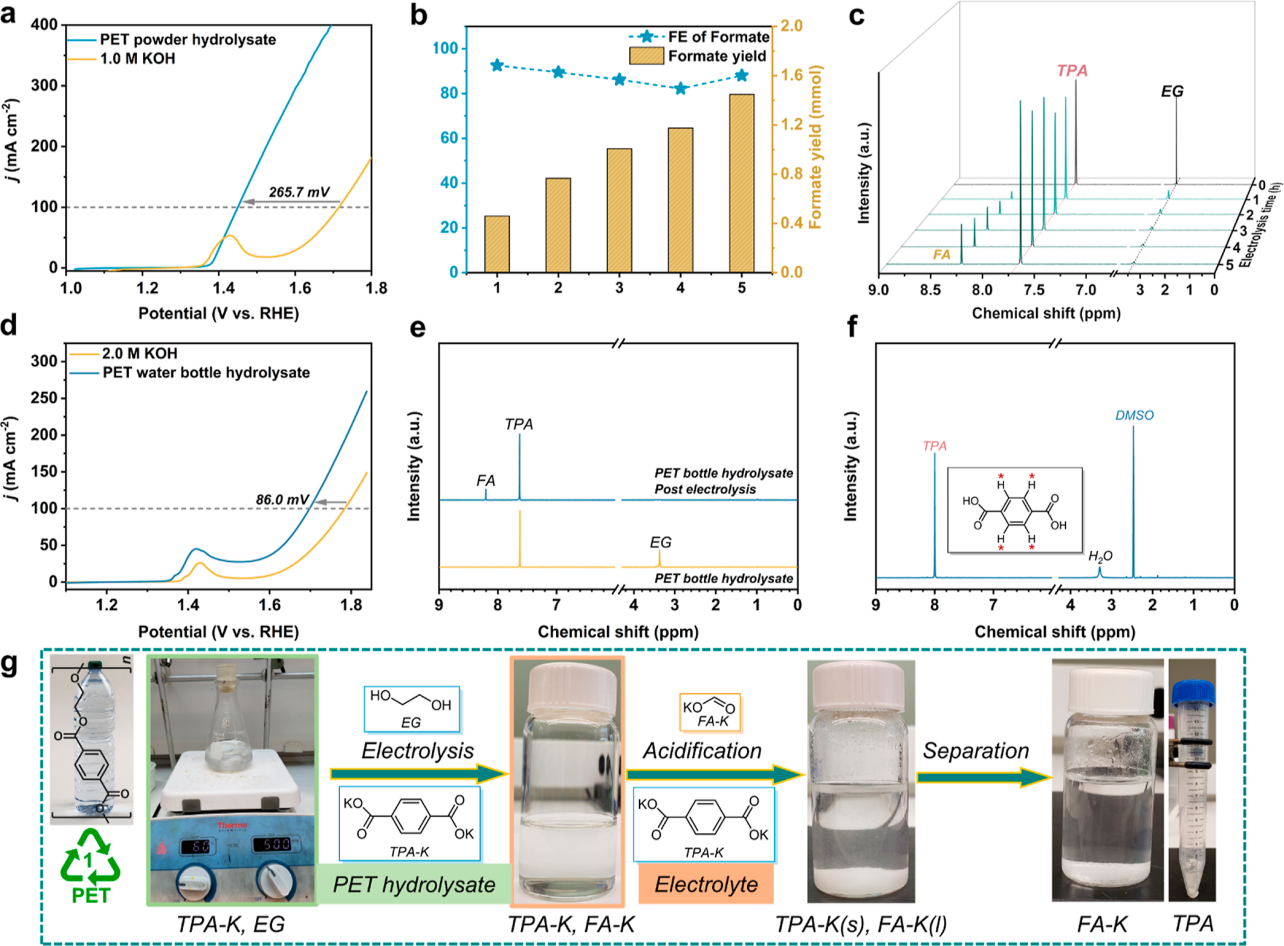
Real-world PET plastic upcycling. (a) LSV curves in PET hydrolysate and 1.0 M KOH. The PET powder hydrolysate was diluted to 1.0 M KOH before electrolysis to avoid the pH difference-induced effect in comparison with the control sample (0.3 M EG). (b) FE and yield of formate with PET powder hydrolysate in a 5 h electrolysis. (c) ^1^H NMR of PET powder hydrolysate electro-oxidation products as a function of electrolysis time. (d) LSV curves of PET water bottle hydrolysate in 2.0 M KOH. (e) ^1^H NMR of Aquafina PET water bottle hydrolysate before and after 1 h electrolysis at 1.52 V vs RHE. (f) ^1^H NMR of terephthalic acid in DMSO recovered from an Aquafina water bottle. (g) Schematic illustration of the electrocatalytic Aquafina water bottle upcycling to formate and TPA.

With a clear electro-oxidation mechanism and an in-depth understanding of the active sites of NiCu_60s_/NF, real-world PET plastic waste upcycling was studied. In this work, commercial PET powder and PET water bottle (Aquafina) were employed as raw materials. PET raw materials were first pretreated in 2.0 M KOH. In the PET powder hydrolysate, TPA and EG were found as the major products by LC–MS (Figure S38) and ^1^H NMR spectroscopy (Figure S40). In addition, a TPA isomer, isophthalate, was also observed. As shown in Figure 6a, when PET powder hydrolysate was applied, the NiCu_60s_/NF electrode displays a 265.7 mV positive potential shift (at 100 mA cm^–2^) compared to the OER. Formate was identified as the only oxidation product with FE over 80% in a 5 h electrolysis (Figures 6b and S40a). In addition, while EG was consumed in the oxidation process, the amount of TPA remained the same throughout the conversion process (Figures 6c and S40b).

To explore the potential practical application of NiCu_60s_/NF, a commercial PET water bottle was employed. As shown in Figure 6d, 86.0 mV of Δ*E* was observed when PET water bottle hydrolysate was utilized, and the smaller Δ*E* compared to PET powder hydrolysate (EG, 174.60 mM) is ascribed to the lower concentration of EG in the PET water bottle hydrolysate (EG, 7.96 mM). Similar to the PET powder hydrolysate, formate was identified as the only oxidation product (Figure 6e), simplifying the separation of the final product separation. Only after 1 h electrolysis (at 1.52 V vs RHE), pure TPA could be recovered by a simple acidification and separation process (Figures S41 and 6g,f).

## Conclusions

In this work, we reported a facile electrodeposition method to synthesize a NiCu/NF catalyst that is capable of upcycling PET efficiently and selectively. The ex situ and in situ experiments indicate that the Cu^0^/CuO core–shell were initially deposited on NF. In the reconstruction process, the original Cu and Ni species were reconstructed into Cu(OH)_2_/CuOOH and NiOOH active species. Compared to NF, the deposited Cu species facilitates the dynamic evolution of catalysts to form active species, promotes faster charger transfer, and increases electrochemically active area, all of which synergistically enhance NiCu/NF’s EGOR performance. Superior formate selectivity (FE, 95.8%) and yield rate (0.70 mmol cm^–2^ h^–1^) were achieved over the optimal NiCu_60s_/NF. In addition, an indirect oxidation mechanism was identified to be the main EGOR mechanism. As well, theoretical calculations revealed that the addition of the Cu species can significantly decrease the Δ*G* value of EGOR’s RDS and facilitates the cleavage of C–C bond, promoting the formation of formate as the sole product and simplifying the end product separation. Lastly, this electrocatalysis system was employed to upcycle real-world PET plastic wastes. With commercial PET water bottle hydrolysate, EG was completely converted to formate with only 1 h of electrolysis, and pure TPA could be recovered via a simple acidification–separation process. This work provides in-depth insights into the dynamic evolution and true active sites of the Ni- and Cu-based electrocatalysts for plastic waste upcycling. It is anticipated to inspire the exploitation of cost-effective catalysts for more efficient plastic waste upcycling.

## Supporting Information Available

SEM, CV curves, and FTIR of Ni foam, Cu foam, and NiCu/NF electrodes; XAS and EDS of NiCu/NF and R-NiCu/NF; extensive discussion of XPS analysis; optimization of electrodeposition time; ^1^H NMR and ^13^C NMR spectra; ECSA measurements; stability tests; in situ Raman analysis; active Cu species identification; indirect oxidation mechanism; and optimized DFT-computed structures (PDF)
