# Supplementary material for: Cu Promoted the Dynamic Evolution of Ni-Based Catalysts for Polyethylene Terephthalate Plastic Upcycling
Source: ACS Catal. 2024 Mar 26;14(7):5314–25. doi: 10.1021/acscatal.3c05509 (PMC11002824; doi:10.1021/acscatal.3c05509)
Supplement: Supplementary file 1 — cs3c05509_si_001.pdf [file cs3c05509_si_001.pdf]

Supporting Information

Cu Promoted the Dynamic Evolution of Ni-based Catalysts for Polyethylene Terephthalate Plastic Upcycling

*Hongxing Kang[a], Dong He[b], Xingxu Yan[c], Benjamin Dao[e], Nicholas B. Williams[a], Gregory I. Elliott[a], Daniel Streater[f], James Nyakuchena[f], Jier Huang[f], Xiaoqing Pan[c,d], Xiangheng Xiao[b], Jing Gu[a],**

[a] Department of Chemistry and Biochemistry, San Diego State University, 5500 Campanile Drive San Diego, CA 92182, USA

[b] Department of Physics, Wuhan University, Wuhan, Hubei 430072, China

[c] Department of Materials Science and Engineering, University of California, Irvine, CA 92697, USA

[d] Department of Physics and Astronomy, University of California, Irvine, Irvine, CA 92697, USA

[e] Department of Chemistry, California State University, Long Beach, Long Beach, CA 90840, USA

[f] Department of Chemistry, Marquette University, Milwaukee, Wisconsin 53201, USA

***Email:** jgu@sdsu.edu

1. Experimental Procedures

1.1 Chemicals

All chemicals were purchased and used as receive unless otherwise stated. Ni foam (NF, 300 mm L × 200 mm W × 1.6 mm T) was purchased from MSE supplies. Cu foam (CF, 480 mm L × 300 mm W × 1.6 mm T) was purchased from MTI Corporation. Copper (II) nitrate hemi(pentahydrate) ($Cu(NO_3)_2 \cdot 2.5H_2O$, 98%) was purchased from Alfa Aesar. Ethylene glycol ($C_2H_6O_2$, 99%) and formic acid (CH_2O_2, 98%) were purchased from Sigma-Aldrich. Glycolic acid ($C_2H_4O_3$, > 98.0%) was purchased from TCI. Glyoxal ($C_2H_2O_2$, 40% w/w aq. soln.) was purchased from Oakwood Chemical. Deuterium oxide (D_2O, D, 99.9 %) and dimethyl sulfoxide-d6 (DMSO-d_6, D, 99.9%) were purchased from Cambridge Isotope Laboratories. Polyethylene terephthalate (PET) powder was purchased from GoodFellow. The PET water bottle was from the commercial Aquafina purified bottled water.

1.2 Preparation of NiCu/NF electrocatalysts

The surface contaminants and oxide layer of NF (1 cm × 2.5 cm) were cleaned by sonication in 4 M HCl for 10 minutes followed by washing with the DI water for 3 times. Then, the NF was dried with nitrogen gas prior to the electrodeposition. The electrodeposition of Cu species on NF (1 cm × 1 cm) was performed at -0.956V (vs. Ag/AgCl) with a potentiostat and a three-electrode setup with a Pt mesh as the counter electrode, an Ag/AgCl as the reference electrode, and NF as the working electrode in 0.1 M $Cu(NO_3)_2 \cdot 2.5\ H_2O$ (pH = 4.15) solution. The electrodeposition time was further optimized based on the electrocatalytic performance of the ethylene glycol oxidation reaction (EGOR). After electrodeposition, NiCu/NF was withdrawn from the electrodeposition solution, rinsed with DI water, and dried at 60°C.

1.3 Reconstruction of NiCu/NF to R-NiCu/NF (EGOR and OER catalysts)

All as-prepared electrodes were activated before EGOR or OER by applying cyclic voltammetry (CV) from 0 to 0.8 V (vs. Ag/AgCl) at 100 mV s^{-1} scan rate for 10 cycles in 1.0 M KOH (pH = 14.00) solution. Herein, a Pt mesh was used as a counter electrode.

To form the reconstructed NiCu/NF (R-NiCu/NF), after CV 10 cycles, one scan of linear sweep voltammetry (LSV) was performed (scan rates of 10 mV s^{-1} from 0 to 0.8 V (vs. Ag/AgCl) in 1.0 M KOH electrolyte). R-NF and R-CF were prepared by the same method as that of the R-NiCu/NF.

1.4 Characterization of electrocatalysts

Powder X-ray diffraction (PXRD) was collected using Bruker D2 Phaser with a Cu Kα source (λ = 1.5418 Å), operated at 30 kV and 10 mA. The acquisitions were carried out at room temperature in the 2θ range of 5° - 80° with a step size of ~0.02°. Each sample was fixed on a piece of pre-cut glass slide (1.5 × 1.5 cm^2) using double-sided carbon tape before loading to a PMMA frontload holder for PXRD analysis.

Fourier transform infrared (FTIR) spectra were obtained using a PERKIN ELMER FTIR spectrometer.

Raman spectra were recorded using a Thermo Scientific DXR Raman microscope with an Ar-ion laser operating at 532 nm. The in-situ Raman measurements were conducted with a homemade in-situ Raman cell (shown in **Figure S28**).

X-ray photoelectron spectroscopy (XPS) spectra were collected using a PHI 5600 XPS machine equipped with an Al Kα X-ray beam (1486.7 eV), running at 250 W and 14 kV. All XPS spectra were corrected by calibrating all peaks to the major adventitious C-C binding energy to 284.8 eV.

Scanning electron microscopy (SEM) analyses were collected using the FEI Quanta 450 FEG SEM at 20 eV.

Scanning transmission electron microscopy (STEM) was performed using JEOL JEM-ARM300CF S/STEM and high-angle angular dark-field (HAADF)-STEM images were acquired at a convergence semiangle of 22 mrad and inner and outer collection angles of 83 and 165 mrad, respectively.

Energy dispersive X-ray spectroscopy (EDS) was conducted using JEOL dual EDS detectors with a specific high count analytical TEM holder.

X-ray absorption spectroscopy (XAS) measurement was performed at 12-BM beamline at the Advanced Photon Source (APS), Argonne National Laboratory. The XAS data were collected under room temperature with fluorescence mode using a 13-element germanium solid-state detector.

1.5 Electrochemical measurements

Electrochemical measurements were performed in an electrochemical cell with a standard three-electrode configuration composed of a Pt mesh as the counter electrode and an Ag/AgCl reference electrode. The as-prepared electrodes were used as working electrode (1 cm × 1 cm in electrolyte). CHI660E was used as the electrochemical workstation. All potentials were recorded with an Ag/AgCl counter electrode and converted to the values referred to the reversible hydrogen electrode (RHE) ($E_{\text{(V vs. RHE)}} = E_{\text{(V vs. Ag/AgCl)}} + 0.197 + 0.059$ pH).

Cyclic voltammetry (CV) was recorded at a scan rate of 100 mV s^{-1}.

Linear sweep voltammetry (LSV) was collected at scan rates of 10 mV s^{-1} without any IR compensation.

Electrochemical impedance spectroscopy (EIS) was conducted at 0.4 V vs. Ag/AgCl over a frequency range from 0.01 Hz to 1 MHz at the amplitude of 5 mV in 1.0 M KOH with/without the addition of 0.3 M EG.

Electrochemically active surface area (ECSA) of electrocatalysts was evaluated by measuring their double-layer capacitance in 1.0 M KOH. The double-layer capacitance (C_{dl}) was derived from CV scans which show a differential capacitance measurement with different scan rates in the non-Faradaic reaction potential range. The C_{dl} was estimated by plotting the $\Delta j = (j_a - j_c)$ at 0.075 V vs. Ag/AgCl (where j_a and j_c are the anodic and cathodic current densities, respectively) against the scan rate, in which the slope is twice that of C_{dl}.

1.6 Electrocatalytic EGOR

EGOR was carried out in an H-typed cell (divided by a Nafion membrane) by Chronoamperometry (i-t) under continuous stirring. Herein, EG was only added into the anodic chamber.

1.7 Chemical oxidation of ethylene glycol

The as-prepared electrodes $NiCu_{60s}$/NF were activated by 10 CV cycles followed by a LSV scan (0 to 0.8 V vs. Ag/AgCl, 10 mV s^{-1}) in 1.0 M KOH solution to form R-$NiCu_{60s}$/NF. After LSV scan, the R-$NiCu_{60s}$/NF was immediately immersed into 1.0 M KOH with the addition of 0.3 M EG.

1.8 EGOR products identification and quantification

The products of EGOR were identified and quantified by ^{1}H NMR based on the standard chemical calibration curves (**Figure S20**). ^{1}H NMR spectra were collected on a 400 MHz Varian spectrometer. The NMR samples were prepared by mixing 540 μL solution after electrolysis and 60 μL D_2O. The water peaks in all ^{1}H NMR spectra were removed from around 4 to 6 ppm by adding a break to clearly demonstrate product peaks.

1.9 Pretreatment of commercial polyethylene terephthalate (PET) powder and water bottle.

6.3 g PET powder (from GoodFellow) was soaked into 100 mL 2.0 M KOH solution in a sealed flask with stirring (500 rpm) for 18 hours at 60 °C. After that, the solid and liquid mixture was separated by centrifuging. The clear aqueous solution was denoted as PET powder hydrolysate and employed for further analysis and PET upcycling.

5 g Aquafina PET water bottle was cut into small pieces and soaked into 100 mL 2.0 M KOH with stirring (500 rpm) for 48 hours at 60 °C. After that, the solid and liquid mixture was separated by centrifuging. The clear aqueous solution was denoted as PET water bottle hydrolysate and employed for further analysis and PET upcycling.

1.10 Products analysis after PET waste pretreatment

The hydrolysis products of PET hydrolysates were further analyzed by ^{1}H NMR and LC-MS.

LC-MS: 100 μL PET powder or PET water bottle hydrolysate was diluted to 10 mL with methanol and analyzed by LC-MS (Agilent 6530B accurate-MASS Q-tof LC/MS)

1.11 Electrochemical upcycling of PET hydrolysate

For LSV, 5 mL PET-hydrolysate was diluted with the addition of 5 mL H_2O (2 M KOH was diluted to 1 M KOH) to exclude the influence of pH differences.

For electrolysis, 8 mL PET-hydrolysate was diluted with 8 mL H_2O (2 M KOH was diluted to 1 M KOH) in the anodic chamber while 16 mL 1.0 M KOH was added into the cathodic chamber. Electrolysis was conducted at 1.47 V vs. RHE for 5 hours. The products were quantified by ^{1}H NMR at each one-hour interval.

1.12 Electrochemical upcycling of PET water bottle.

PET water bottle hydrolysate was directly used as substrate without dilution. After electrolysis at 1.52 V vs. RHE for 1 h, the anodic solution was concentrated by the rotary evaporator and acidified with pure formic acid to result in the precipitation of TPA. Further, the solid and liquid parts were isolated by centrifuging. The solid product was dried in an oven at 60 °C. Following that, the solid product was dissolved into DMSO-d_6 for the NMR analysis.

1.13 Calculation Equations

The Faradaic efficiency (FE) and yield rate were calculated using the following equations.

$$FE(\%) = \frac{mole\ of\ products}{total\ charge\ passed/(n \times 96485\ C\ mol^{-1})}$$

$$Formate\ yield\ rate\ (mmol\ cm^{-2}h^{-1}) = \frac{amount\ of\ produced\ formate\ (mmol)}{electrode\ area(cm^2) \times reaction\ time\ (h)}$$

Where n is numbers of electron transferred for each product. Herein, n is 3 for formate, while it is 4 for glycolate. 96485 C mol^{-1} is used as the Faraday constant.

The moles of products were calculated based on the standard chemicals ^1H NMR calibration curves as shown in **Figure S20**.

1.14 Computational methods

All calculations were performed using the CASTEP Package. The GGA-PBE exchange-correlation potential and ultrasoft pseudopotentials were used. The energy cutoff was set to 450.0 eV and the 2 × 2 × 1 MonkhorstPack mesh k-point was employed for the surface calculations.

The DFT+D dispersion correction was employed to account for the Van Der Waals (vdW) interactions. The (010) crystal plane of NiOOH is first constructed to ensure that Ni atoms located at the octahedral position serve as the effective adsorption sites for reaction intermediates. To simulate NiCu/NF active species, a layer of NiOOH was replaced by a layer of CuOOH. At the same time, a 15a Å vacuum layer was constructed to ensure that the adsorbent is not disturbed in the Z direction. The convergence tolerances were set to 2×10^{-5} eV per atom for energy, 2×10^{-3} Å for maximum displacement, and 0.05 eV $Å^{-1}$ for maximum force during the geometry optimization. In the entire geometry optimization, the bottom three layers of atoms are fixed to simulate the bulk phase of the crystal.

The Gibbs free energy barrier (ΔG) calculations of each elementary step were based on the calculated hydrogen electrode model proposed by Nørskov et al.,[1] which can be determined as

$\Delta G = \Delta E + \Delta E_{ZPE} - T \cdot \Delta S$

where ΔE and ΔS are the reaction energy and entropy change and ΔE_{ZPE} is the difference in zero-point energy. The ZPE and entropic corrections were performed

through frequency calculations. At the same time, all data in our article were discussed in the case of V vs. SHE = 0 V.

2. Results and Discussion

2.1 Structures and Morphologies

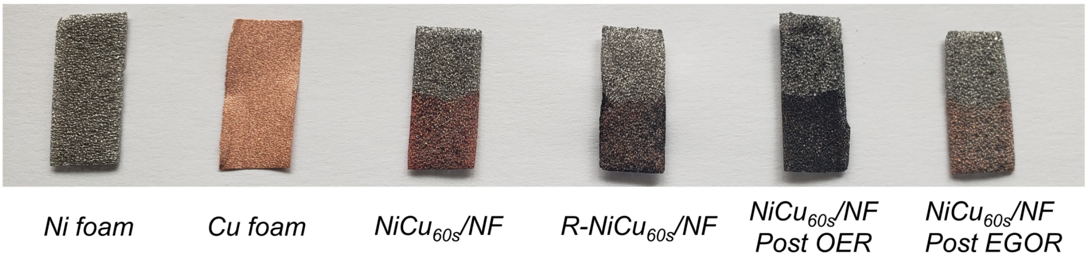

Figure S1. The optical photography of the prepared electrodes, where $NiCu_{60s}/NF$ represents Cu species deposited on a NF electrode after 60 s. R-$NiCu_{60s}/NF$ represents the catalyst after reconstruction.

Post OER sample ($NiCu_{60s}/NF$-Post OER) was received by electrolysis at 1.62 V vs. RHE for 2 h in 1.0 M KOH solution.

Post EGOR sample ($NiCu_{60s}/NF$-Post EGOR) was received at 1.42 V vs. RHE for 2 h in 1.0 M KOH with the addition of 0.3 M EG.

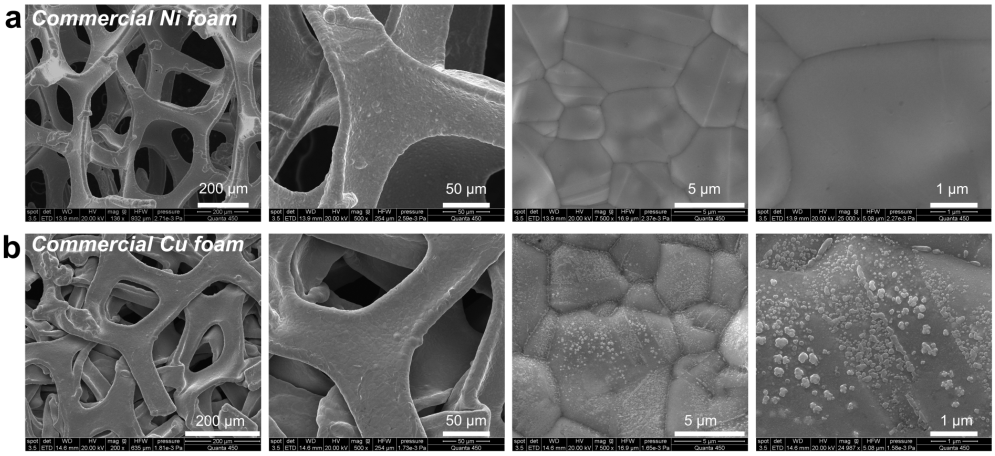

Figure S2. SEM images of (a) commercial Ni and (b) Cu foams. Prior to the SEM characterizations, Ni foam, and Cu foam were cleaned by sonicating in 4 M HCl for 10 mins to remove the oxidized layer and contaminants.

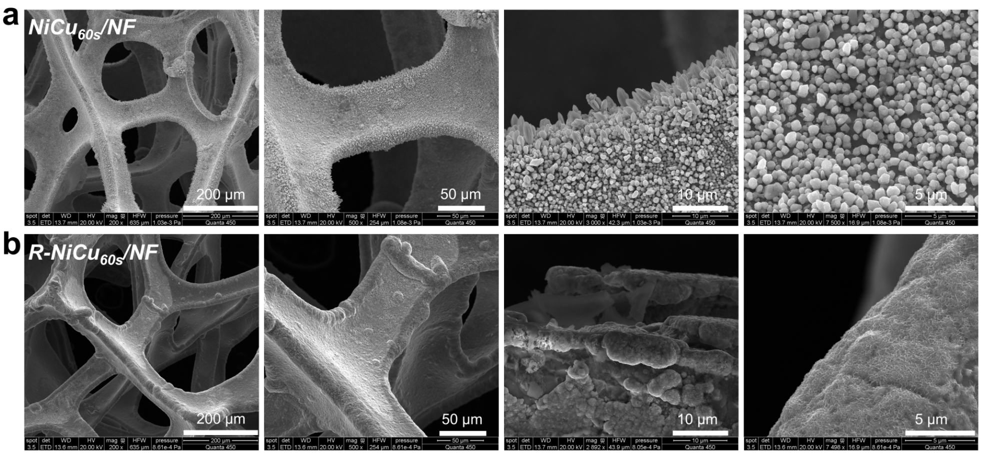

Figure S3. SEM images of (a) the prepared $NiCu_{60s}/NF$ and (b) reconstructed $NiCu_{60s}/NF$ (R-$NiCu_{60s}/NF$).

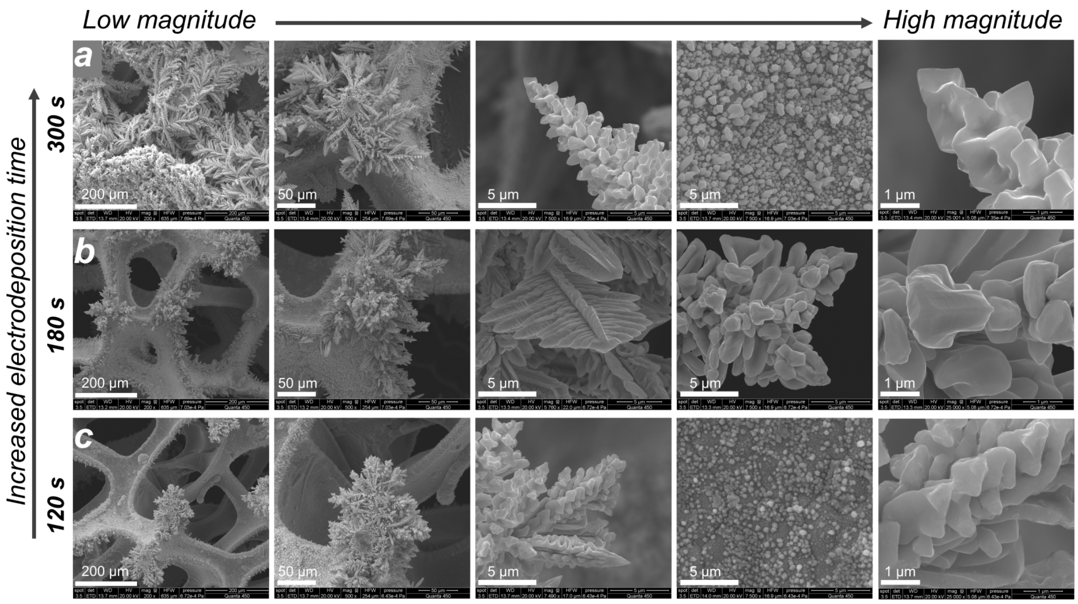

Figure S4. SEM images of prepared $NiCu_{60s}/NF$ with different electrodeposition times (a) 300 s, (b) 180 s, and (c)120 s.

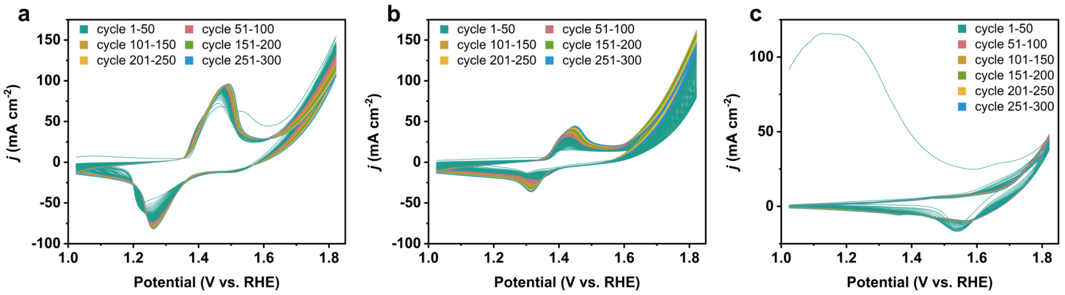

Figure S5. CV scans of (a) $NiCu_{60s}/NF$, (b) Ni foam, and (c) Cu foam in 1.0 M KOH at a scan rate of 100 mV s^{-1}.

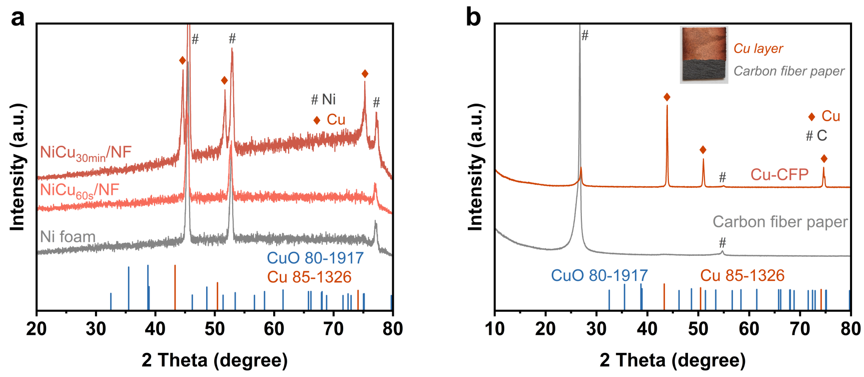

Figure S6. XRD patterns of Cu electrodeposition on (a) Ni foam and (b) carbon fiber paper.

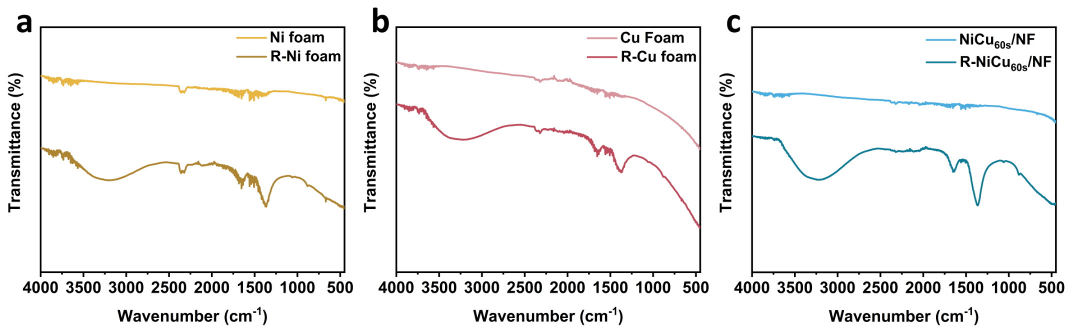

Figure S7. FTIR spectra of (a) Ni foam, (b) Cu foam, and (c) $NiCu_{60s}/NF$ before and after the reconstruction process.

R-Ni foam, R-Cu foam, and R-$NiCu_{60s}/NF$ represent Ni foam, Cu foam, and $NiCu_{60s}/NF$ after the reconstruction process, respectively (**Figure S7**). Herein, all electrodes display three similar peaks. The absorption band at 1653 cm^{-1} is attributed to the bending vibration of water adsorbed on the electrodes' surface. Additionally, the bands at 3221 cm^{-1} and 1362 cm^{-1} can be assigned to -OH stretching and deformation vibrations, respectively. These characteristic peaks correspond to the formation of metal hydroxides after the reconstruction process.[2]

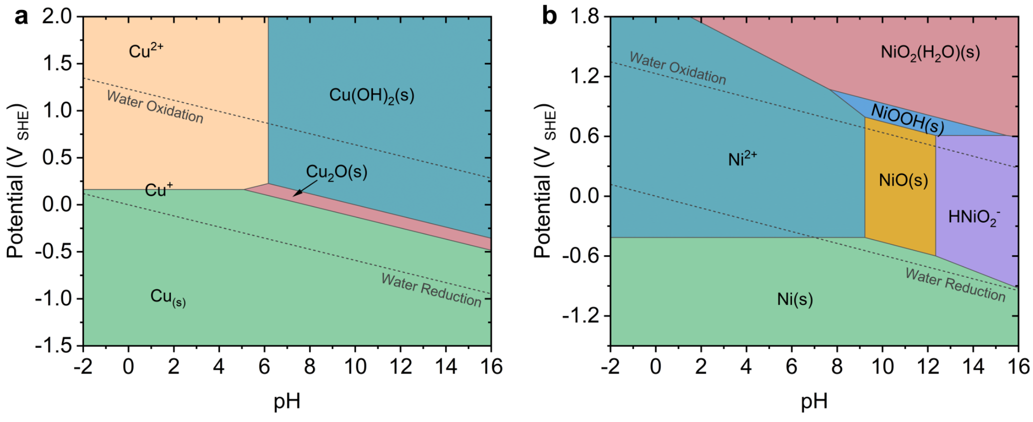

Figure S8. Simulated Pourbaix diagrams of (a) Cu and (b) Ni at 298.15 K using Factsage software.[3] The molality of aqueous species was 1×10^{-6} mol/kg.

2.2 X-ray photoelectron spectroscopy (XPS) analysis section

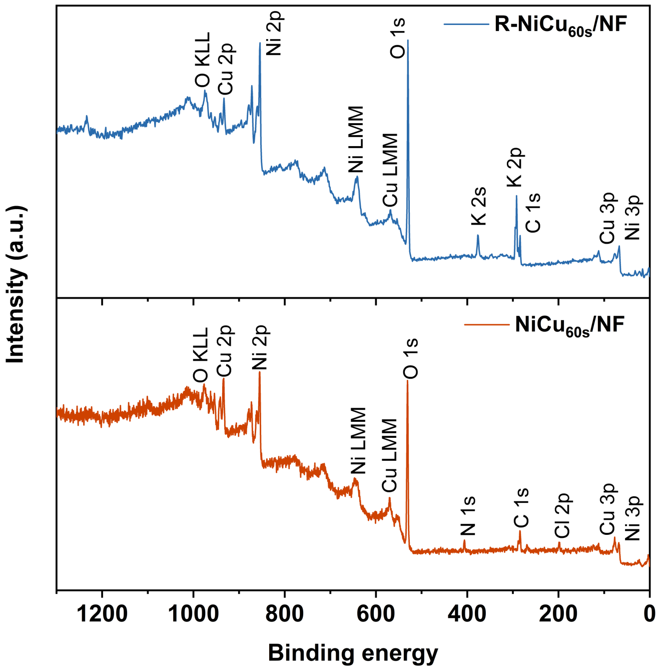

Figure S9. XPS survey spectra of $NiCu_{60s}$/NF and R-$NiCu_{60s}$/NF.

The survey scan of $NiCu_{60s}$/NF (**Figure S9**) revealed the presence of Cu, Ni, O, C, N, Cl and C elements. The presence of Cl is attributed to the use of Ag/AgCl reference electrode in the electrodeposition process. For the reconstructed sample, R-$NiCu_{60s}$/NF, the presence of K might originate from the KOH electrolyte.

The impact of air oxidation on metallic species.

All electrodes were prepared by electrodepositing at -0.956 V vs. Ag/AgCl in 0.1 M $Cu(NO_3)_2{\cdot}2.5\ H_2O$ solution (pH, 4.15). Under this condition, Cu^{2+} ions will be reduced to Cu^0 and deposited on Ni foam according to the Pourbaix diagram (**Figure S8a**).[4] However, all ex-situ results indicate the existence of CuO species on the surface. Therefore, it is reasonable to assume the deposited metallic Cu^0 could be oxidized to CuO when exposed to air and forming a Cu/CuO core-shell structure.

To confirm this, XPS measurements and analysis were further performed.

The importance of shake-up satellite peak in Cu species XPS spectrum.

It is necessary to mention the importance of shake-up satellite peaks in Cu 2p XPS spectrum in identifying the paramagnetic Cu^{2+} species (d^9 configuration).

As pointed out by the work of Mark C. Biesinger,[5] shake-up satellite peaks may occur when the outgoing photoelectron simultaneously interacts with a valence electron, exciting it to a higher-energy level. Following that, the kinetic energy of the core electron will be slightly reduced, according to the equation: $KE = h\nu - BE - \Phi$, giving a satellite peak at a higher binding energy than the main lines.

As a classic example, shake-up peaks are present in the $2p_{3/2}$ spectra of d^9 Cu(II) species ($Cu(OH)_2$ and CuO) but are absent in the d^{10} Cu (0) or Cu(I) (Cu_2O) spectra.[6] Therefore,

the presence of satellite peak is an important sign to qualitative or semi-quantitatively identify the Cu^{2+} species.[5]

XPS experimental section

To illustrate the effects of air oxidation during the characterization processes, two samples, $NiCu_{60s}$/NF and $NiCu_{180s}$/NF, where $NiCu_{60s}$/NF and $NiCu_{180s}$/NF were prepared by electrodepositing for 60 s and 180 s, respectively. $CuNi_{60s}$/NF and $CuNi_{180s}$/NF samples were dried under 60 °C for 3 h and 45 h prior to the XPS measurements.

After exposed to air for 3 h, in the Cu $2p_{3/2}$ spectrum (**Figure S10a)** of $CuNi_{60s}$/NF, Cu^{2+} (933.6 eV) and Cu^{+}/Cu^{0} (932.5 eV) species were identified while only Cu^{+}/Cu^{0} (932.7 eV) can be curve-fitted in the $CuNi_{180s}$/NF sample. Meanwhile, smaller satellite peaks were observed in both samples, suggesting the presence of CuO. It is worth mentioning that Cu^{2+} is present in $CuNi_{180s}$/NF even though its Cu $2p_{3/2}$ cannot be fitted. This is ascribed to the amount of oxidized Cu^{2+} is relatively smaller compared to Cu^{+}/Cu^{0} in the $CuNi_{180s}$/NF, causing the fitting of Cu $2p_{3/2}$ peak by Cu^{2+} and Cu^{+}/Cu^{0} to be difficult.

In comparison with air exposure for 3 h, stronger satellites, and a much higher $Cu^{2+}/(Cu^{1+}+Cu^{0})$ ratio were observed when both samples were exposed to air for 45 h. Mark C. Biesinger developed a mathematical method to quantitatively determine the ratio of Cu^{0}/Cu^{2+} and Cu^{+}/Cu^{2+}.[5] Based on this method, the percent of CuO species can be derived by calculating the ratio of the satellite peak area to the total peak area (**Table S3)**. Specifically, the percent of CuO increases from 10.63% to 34.43% and 0.49% to 19.10% in the $CuNi_{60s}$/NF and $CuNi_{180s}$/NF, respectively, when the air exposure time was increased from 3 h to 45 h. This result suggests that air oxidation is the main reason for the formation of CuO. In addition, when the air exposure time was 3 h, Ni^{0} species was observed in the $NiCu_{60s}$/NF while it was absent in the $NiCu_{180s}$/NF, possibly resulting from the coverage of thicker Cu overlayer due to the longer electrodeposition time. In addition, the increased M-O species percent in the O 1s spectra when the air exposure time was increased (3 h to 45 h) further supports the fact that Cu can be spontaneously oxidized to CuO in air.

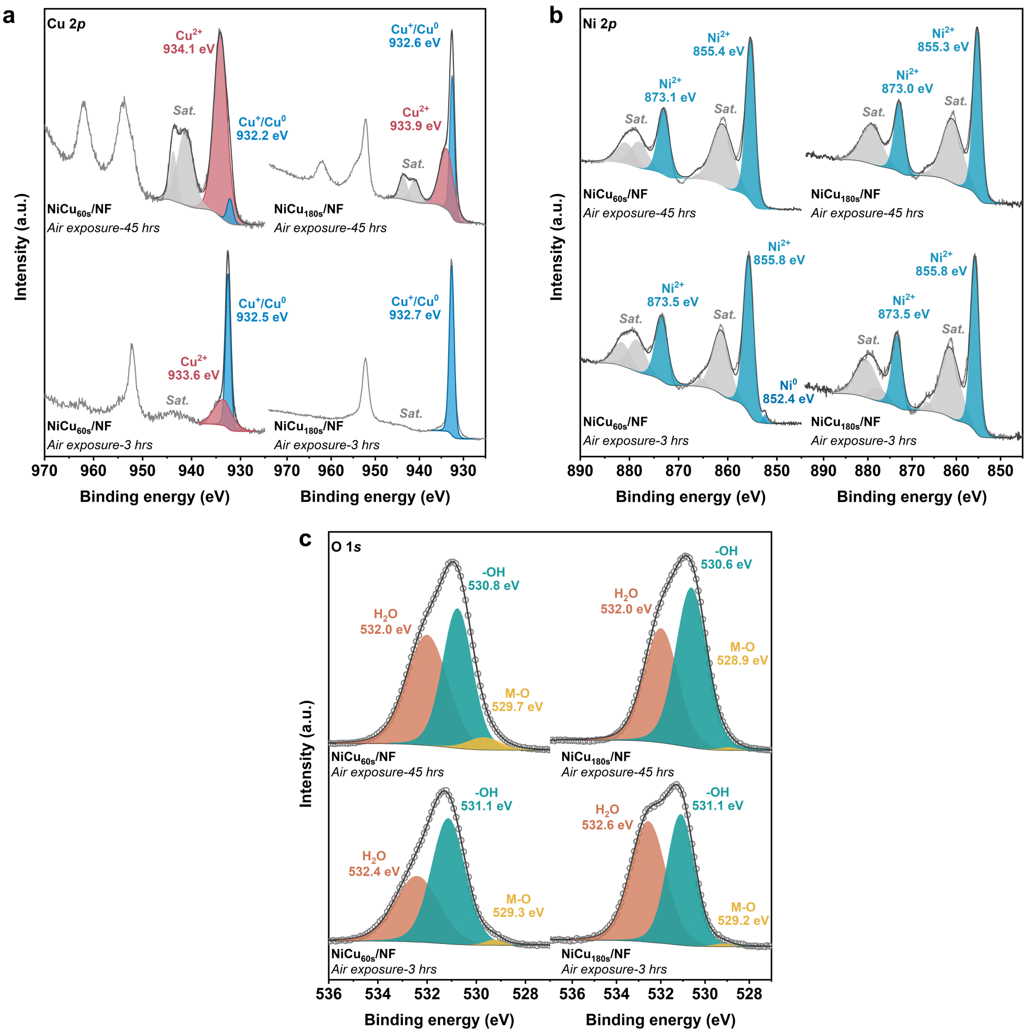

Figure S10. (a) Cu 2p, (b) Ni 2p and (c) O 1s XPS spectra of $NiCu_{60s}$/NF and $NiCu_{180s}$/NF after 3 h and 45 h exposing to air, respectively.

XPS depth-profiling analysis

To confirm the formation of the core-shell structure and estimate the depth of the oxidized layer (shell), Ar^+ depth profile was conducted with the following specifications: Ar^+ ion source operates with the 2 kV Ar^+ beam energy and the sample slit was set to 30 degrees. Sample raster and sputter rate were set to 7.5 mm × 7.5 mm and 0.01 nm/min, respectively.

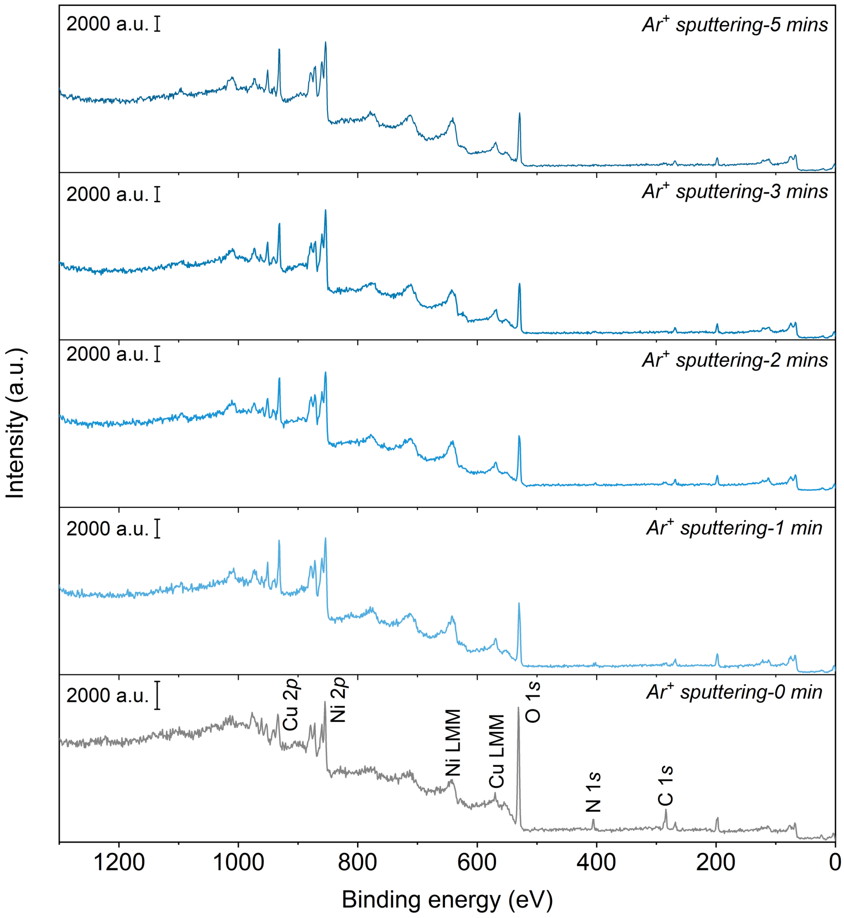

Figure S11. XPS survey spectra of $NiCu_{60s}$/NF (exposed to air for 45 h) as a function of Ar^+ sputtering time from 0 to 5 mins.

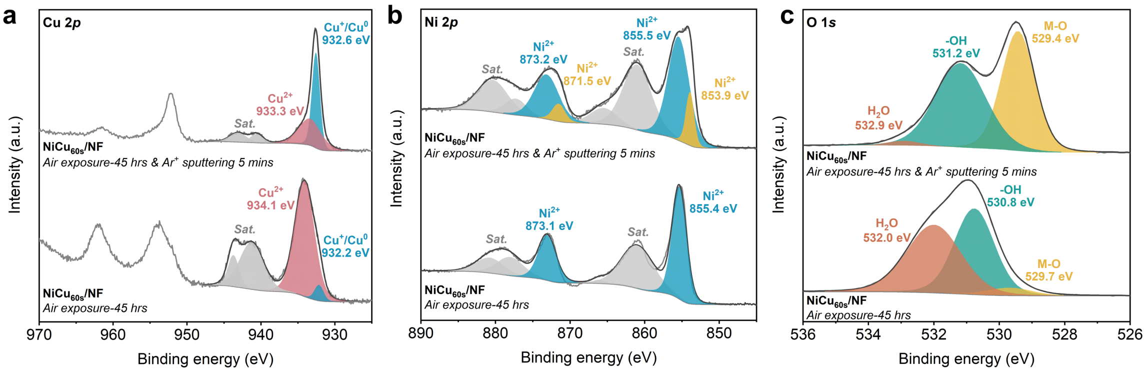

Figure S12. (a) Cu 2p, (b) Ni 2p, and (c) O 1s XPS spectra of $NiCu_{60s}$/NF (exposed to air for 45 h) before and after 5 min Ar^+ sputtering.

As shown in **Figure S11,** with an increased time (0 to 5 min) of Ar^+ sputtering, O 1s peak intensity was apparently reduced, suggesting the removal of the oxidized metal species from the electrode's surface.

As displayed in **Figure S12** and **Table S4**, for Cu 2p spectra, after 5 mins of Ar^+ sputtering, the percent of CuO species was reduced from 34.43% to 15.32% (calculated from satellite peak area to total peak area) and a higher percentage of Cu^+/Cu^0 was observed.

Meanwhile, NiO (at 853.9 eV) species were observed from the Ni 2p spectra after Ar^+ sputtering, indicating the NiO layer is under the $Ni(OH)_2$ (at 855.4 eV) layer. Ar^+ depth profile sputtering results indicate that the metal on the electrodes' surface was oxidized to higher oxidation states when the sample was exposed to air while the core metals remained at their lower oxidation states.

Ar^+ sputtering for the reconstructed samples

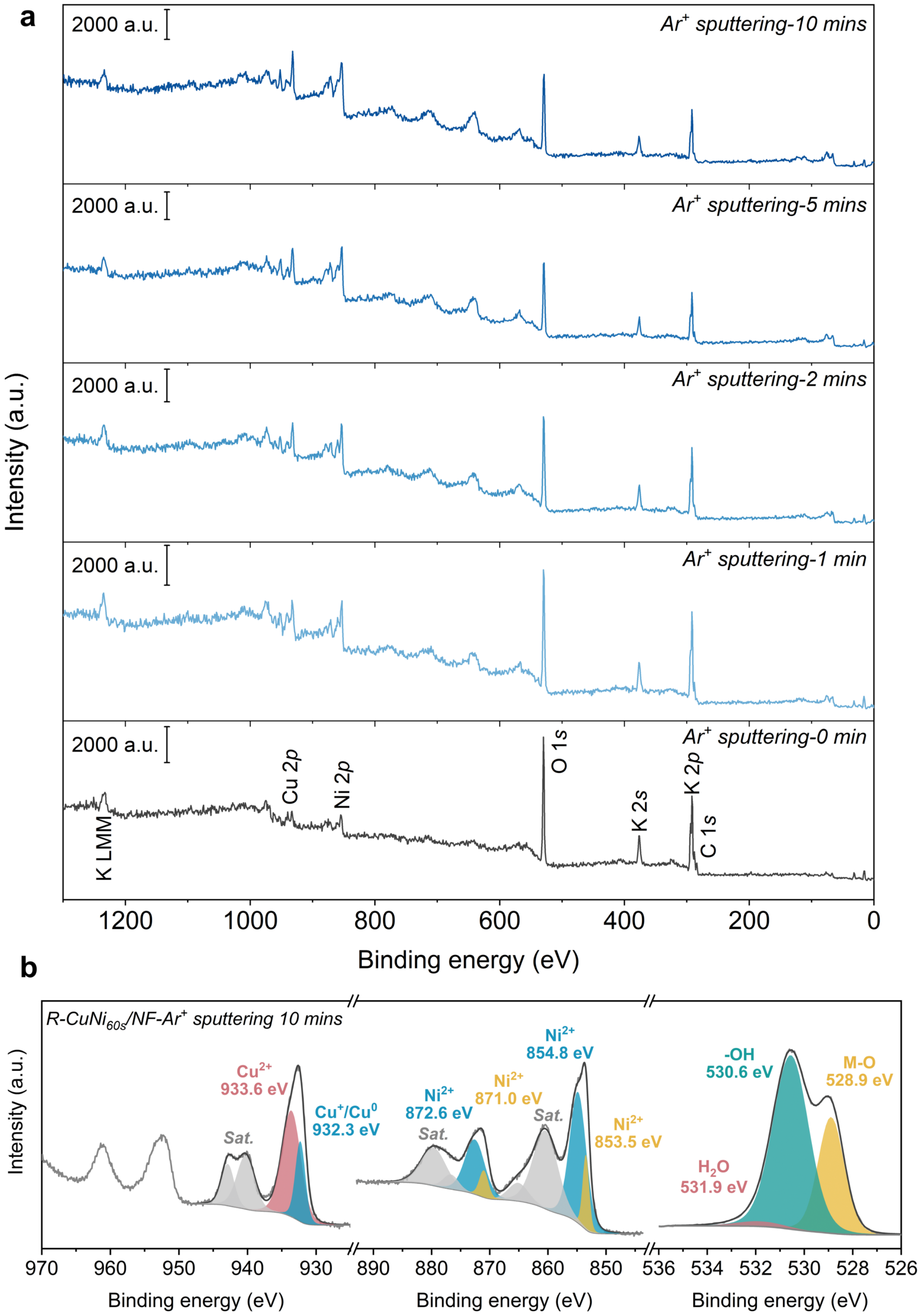

Figure S13. XPS survey spectra of R-NiCu$_{60s}$/NF (a) as a function of Ar^+ sputtering time from 0 to 10 min and (b) Cu 2p, Ni 2p, and O 1s spectra after 10 min Ar^+ sputtering.

To investigate oxidation states of the core metals after reconstruction, 10 mins of Ar^+ sputtering was further conducted on the R-$NiCu_{60s}$/NF sample to remove the surface oxide/hydroxide layer. As shown in **Figure S13b**, after 10 mins of Ar^+ sputtering, low-oxidation state Cu species and NiO were present, which indicates the surface metals were reconstructed to their oxidized forms as CuO and $Ni(OH)_2$ while the core metals remain in the Cu^0 and NiO forms.

2.3 X-ray absorption spectroscopy (XAS) analysis

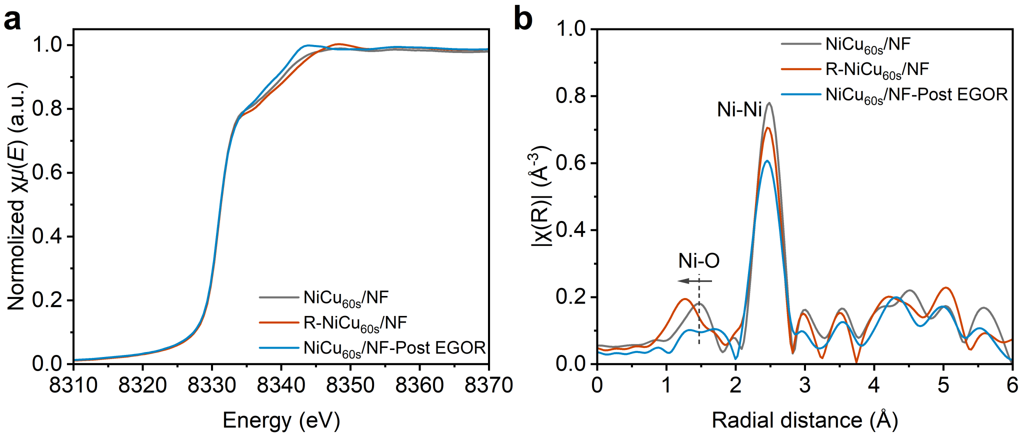

Figure S14. (a) Normalized Ni K-edge XANES spectra and (b) k^2-Weighted Ni K-edge FT-EXAFS spectra of $NiCu_{60s}$/NF, R-$NiCu_{60s}$/NF, and $NiCu_{60s}$/NF after EGOR.

As shown in **Figure S14a,** after EGOR in R-$NiCu_{60s}$/NF, the Ni K-edge shifted to a lower energy direction compared to that of $NiCu_{60s}$/NF, indicating a decreased Ni oxidation state. Meanwhile, the weaker Ni-O peak intensity in $NiCu_{60s}$/NF-post EGOR (**Figure S14b**) also implies that Ni has a reduced oxidation state after reconstruction.

2.4 Energy dispersive X-ray spectroscopy (EDS) analysis

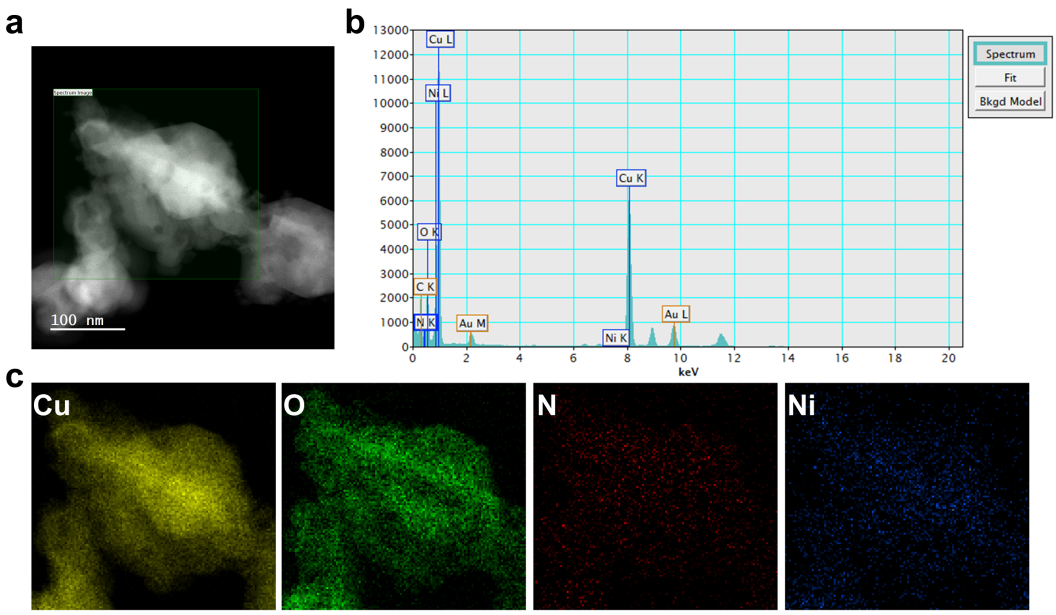

Figure S15. EDS analysis of NiCu/NF. (a) STEM image of the NiCu/NF. The green line squared area was selected as the EDS scanning area. (b) The EDS spectrum of existing elements in the sample. Herein, Au originated from Au TEM grids. (c) The corresponding elemental mapping. In this NiCu/NF sample, the electrodeposition time was set to 30 mins in order to collect enough catalyst for analysis.

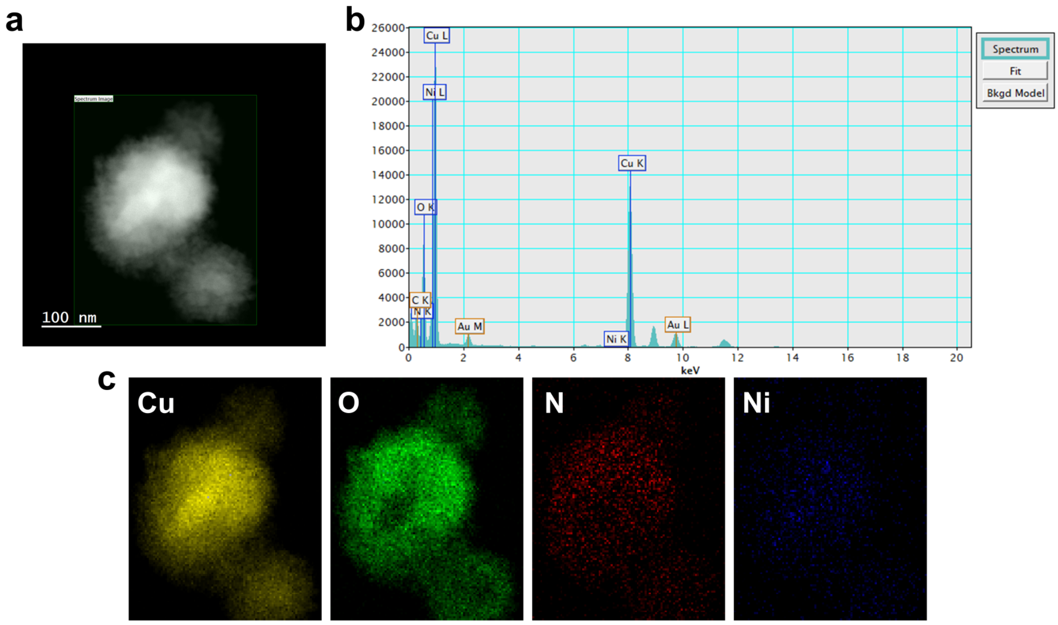

Figure S16. EDS analysis of R-NiCu/NF. (a) STEM image of the NiCu/NF for EDX analysis. The green line squared area was selected as the EDS scanning area. (b) The EDS spectrum of the existing elements in the sample. Herein, Au originated from the Au TEM grids. (c) The corresponding elemental mapping. In this R-NiCu/NF sample, the electrodeposition time was set to 30 mins in order to collect enough catalyst for analysis.

2.5 Electrooxidation of ethylene glycol (EGOR)

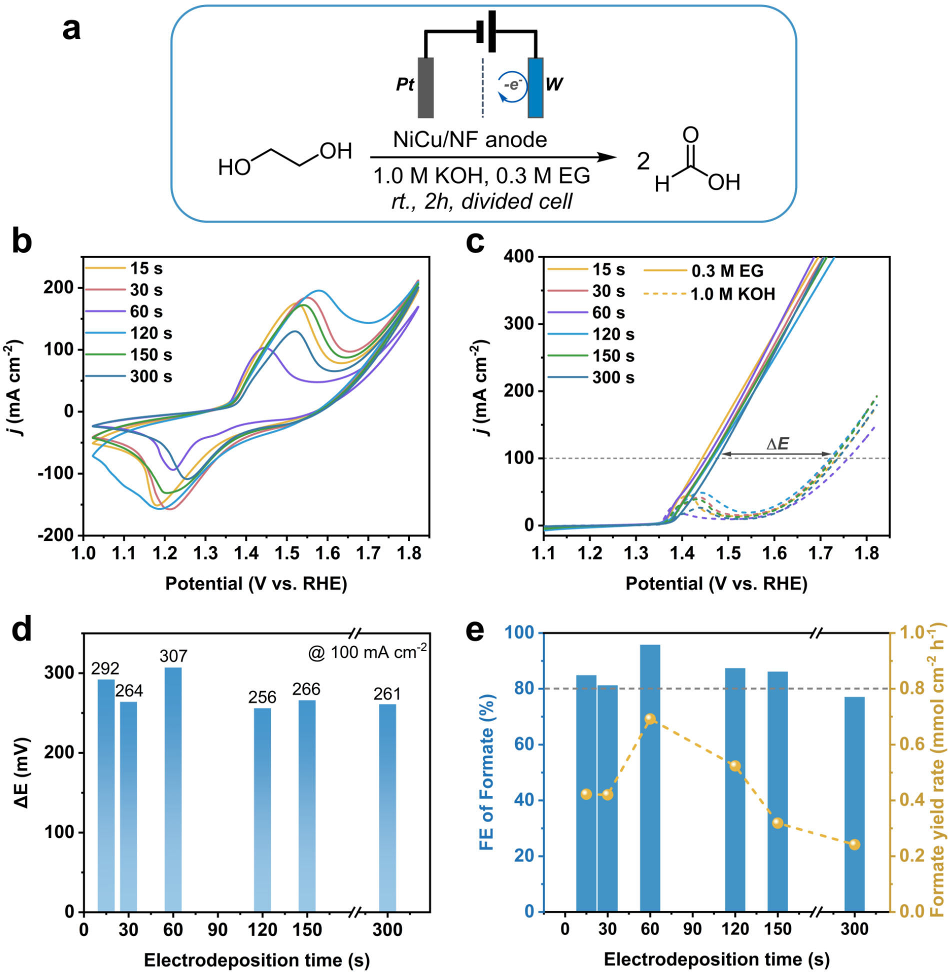

Figure S17. (a) Illustration of EGOR in a divided cell (b) CV curves of NiCu/NF with varied electrodeposition times in 1.0 M KOH. (c) LSV curves of NiCu/NF with different electrodeposition times in OER (1.0 M KOH) and EGOR (0.3 M EG) conditions, respectively (d) ΔE values of different NiCu/NF catalysts, where ΔE represents the potential difference between EGOR and OER at 100 mA cm^{-2}. (e) FEs and yield rates of formate after 2 h electrolysis of NiCu/NF with different electrodeposition times at 1.47 V vs. RHE. Herein, the optimal electrodeposition time was determined to be 60 s.

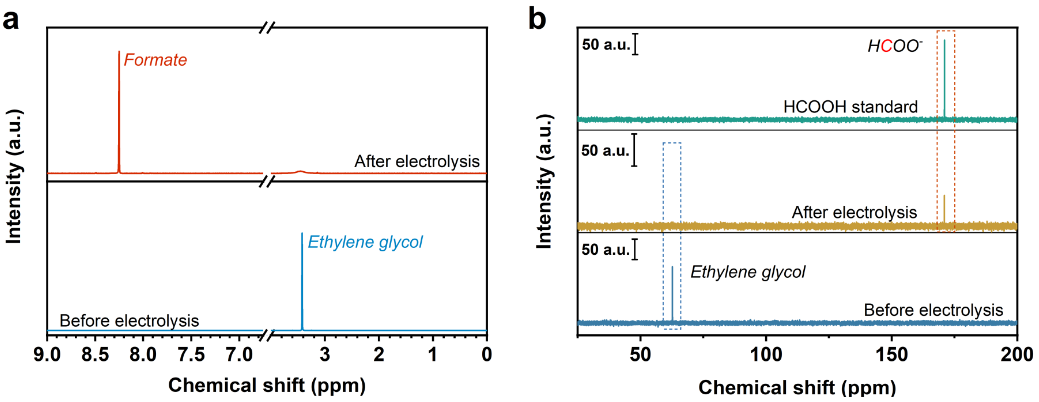

Figure S18. (a) ^{1}H NMR and (b) ^{13}C NMR before and after electrolysis of 0.3 M EG in 1.0 M KOH for 24 h at 1.47 V vs. RHE, the concentration of HCOOH standard was 500 mM.

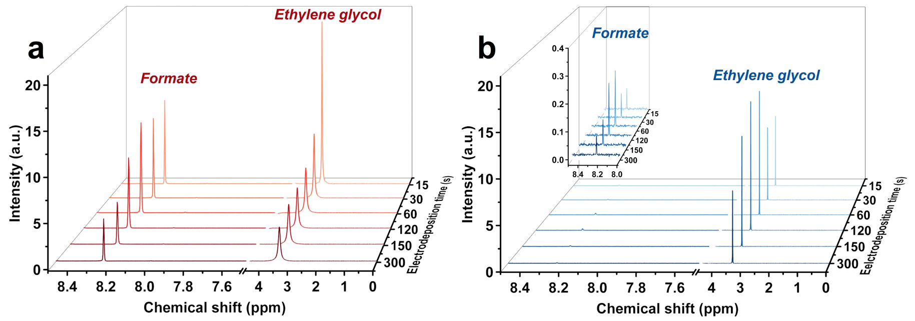

Figure S19. ^{1}H NMR spectra of (a) anodic and (b) cathodic chamber products of NiCu/NF with different electrodeposition times. Electrolysis conditions: at 1.47 V vs. RHE for 2 h in 0.3 M EG solution.

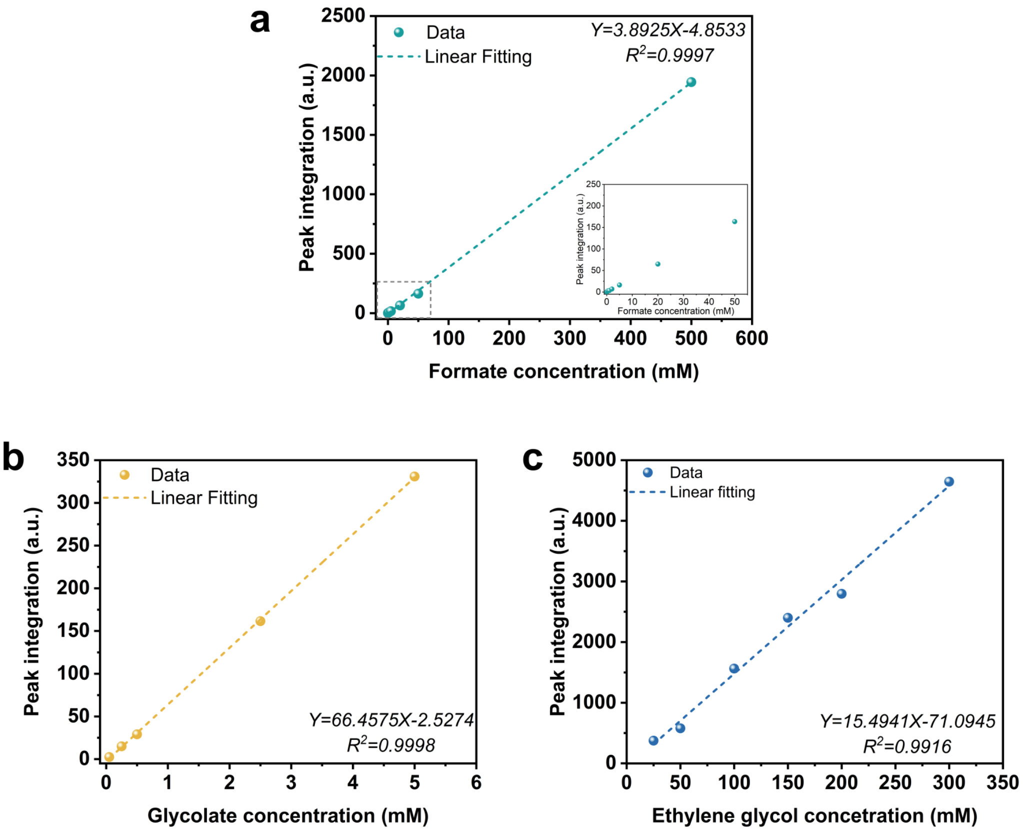

Figure S20. Calibration curves of (a) formate, (b) glycolate, and (c) ethylene glycol in 1.0 M KOH.

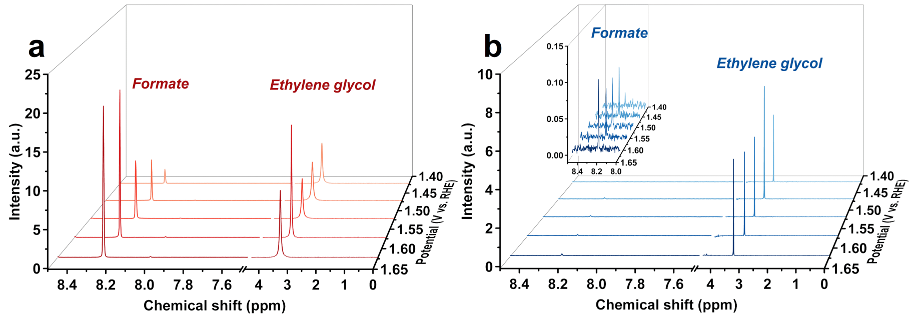

Figure S21. Ethylene glycol potential-dependent electrolysis of $NiCu_{60s}$/NF electrode. ^{1}H NMR spectra of (a) anodic and (b) cathodic chamber products at different potentials. Electrolysis conditions: electrolysis of 0.3 M EG in 1 M KOH solution for 1 h.

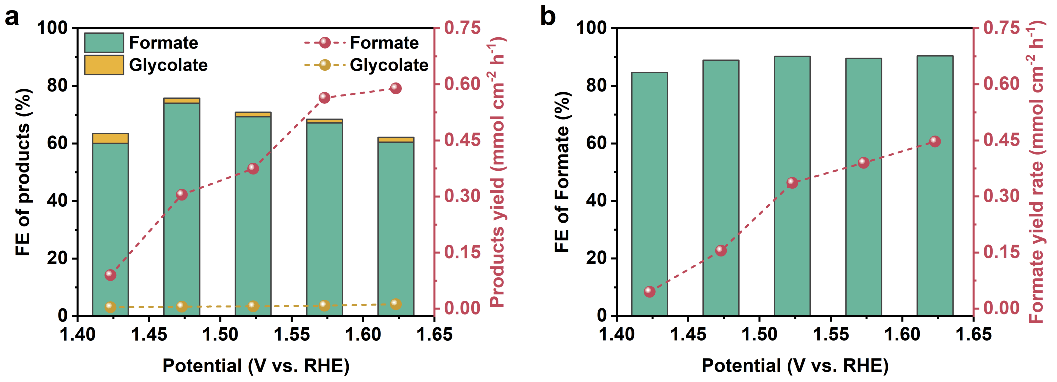

Figure S22. Products FEs and yield rates of (a) Ni foam and (b) Cu foam electrodes for EGOR over 1 h electrolysis at different applied potentials.

ECSA measurements

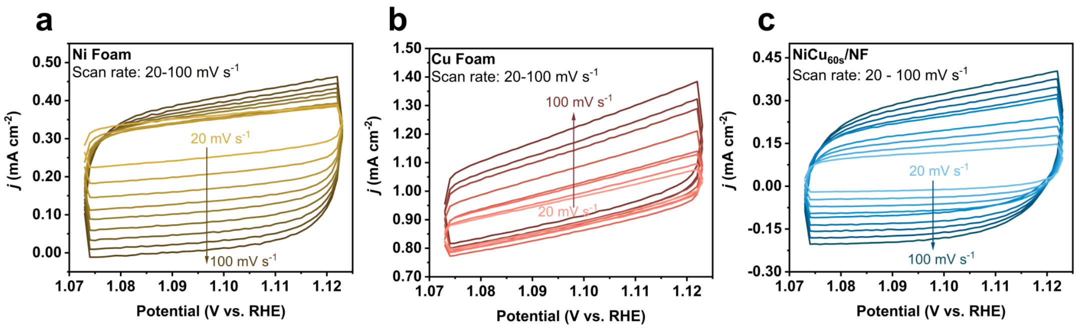

Figure S23. Electrochemical active surface area (ECSA) analysis of (a) Ni foam, (b) Cu foam, and (c) $NiCu_{60s}$/NF electrodes at varied scan rates from 20 mV s^{-1} to 100 mV s^{-1} in 1.0 M KOH.

2.6 Electrocatalyst stability evaluation

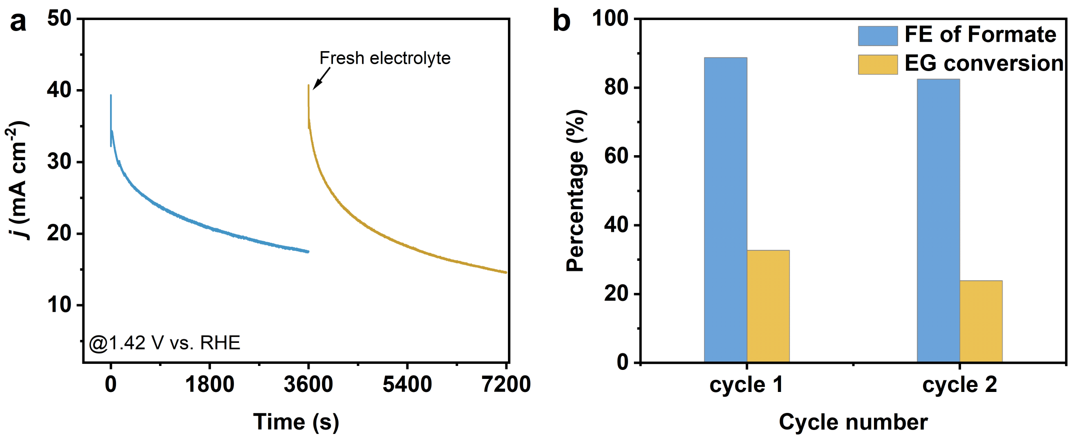

Figure S24. (a) Chronoamperometry at 1.42 V vs. RHE in two successive electrolysis cycles in 0.3 M EG solution. Fresh EG solution was injected after first cycle to re-establish the initial EG concentration and (b) FE of formate and EG conversion for each electrolysis cycle at 1.42 V vs. RHE.

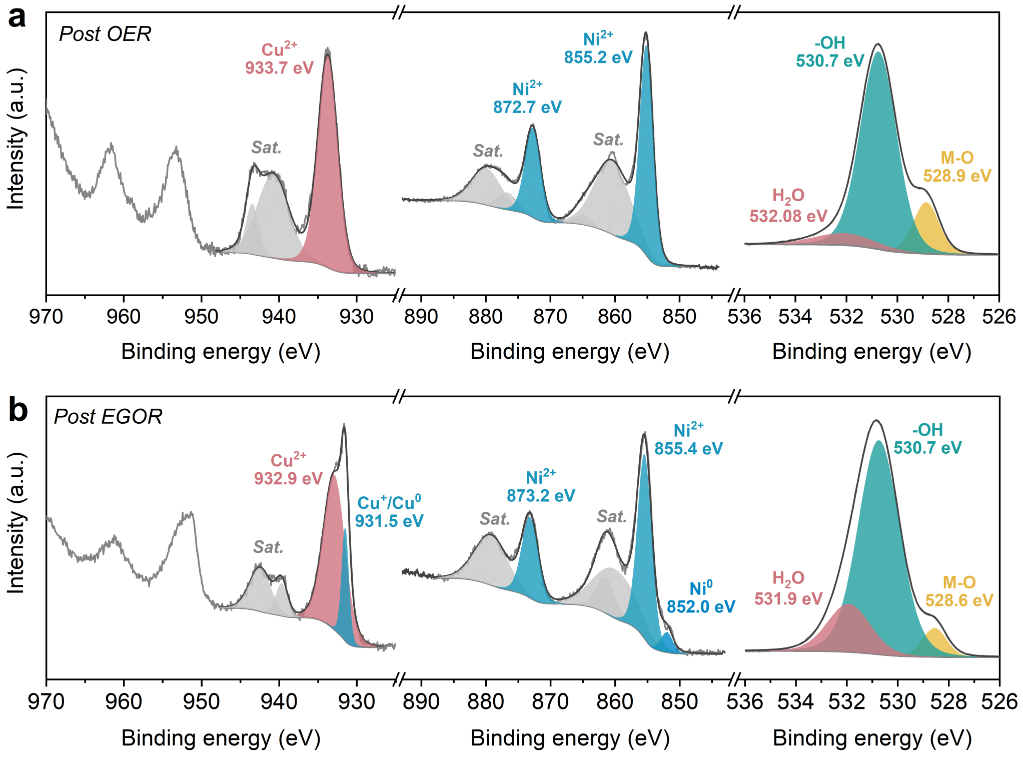

Figure S25. Cu 2p, Ni 2p, and O 1s XPS spectra of $NiCu_{60s}$/NF after OER (a) and EGOR (b), respectively.

OER condition: at 1.62 V vs. RHE for 2 h in 1.0 M KOH solution

EGOR condition: at 1.42 V vs. RHE for 2 h in 1.0 M KOH with the addition of 0.3 M EG.

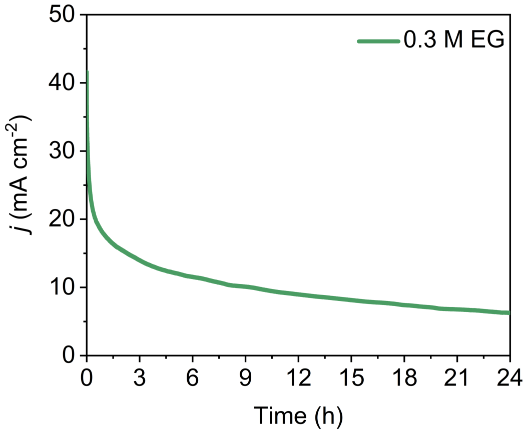

Figure S26. Chronoamperometry at 1.42 V vs. RHE for 24 h in 0.3 EG solution. In the 24 h stability test, a significant decrease in current density was observed, This phenomenon was mainly attributed to the consumption of EG.

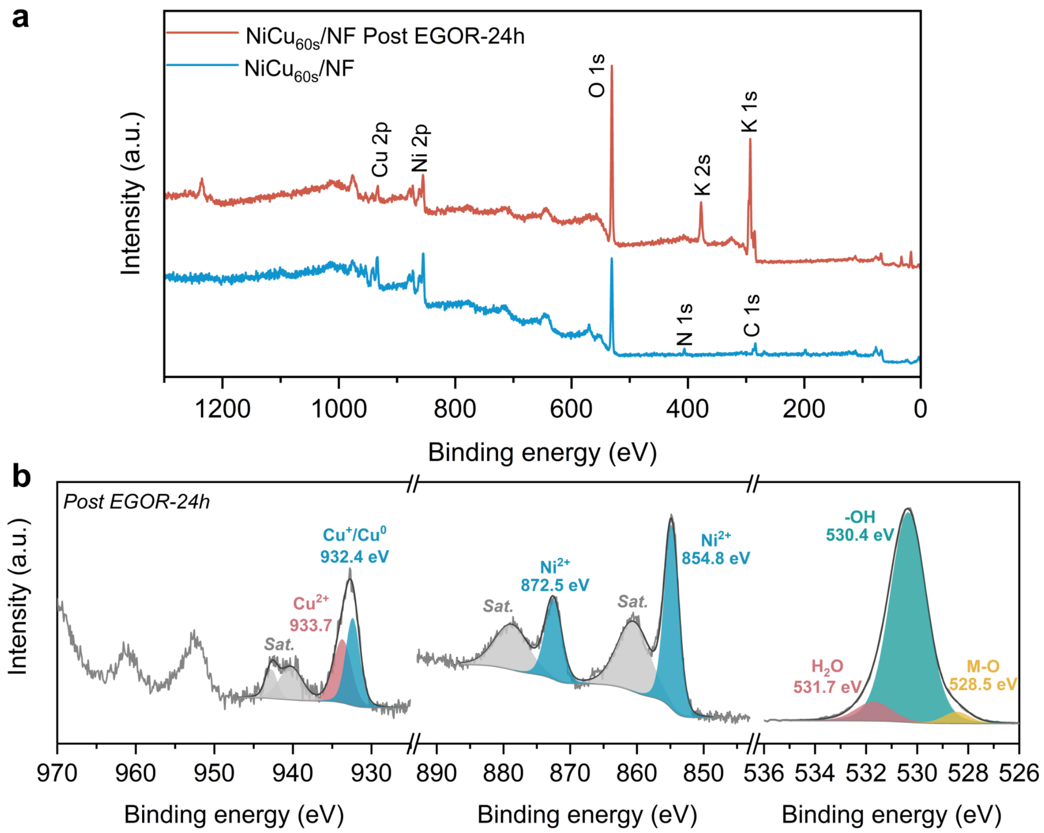

Figure S27. (a) XPS survey spectra of $NiCu_{60s}$/NF before and after 24-hour EGOR. (b) Cu 2p, Ni 2p, and O 1s of $NiCu_{60s}$/NF after 24 h EGOR (at 1.42 V vs. RHE).

In-situ Raman cell

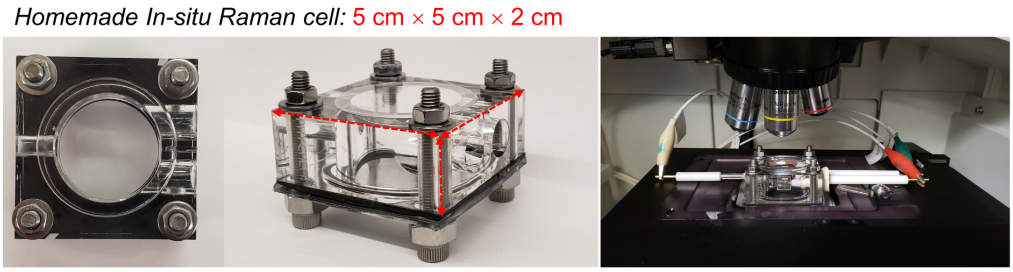

Figure S28. Optical photography of the homemade in-situ Raman cell.

2.7 Cu active species identification section

CV studies of the Cu species on different substrates

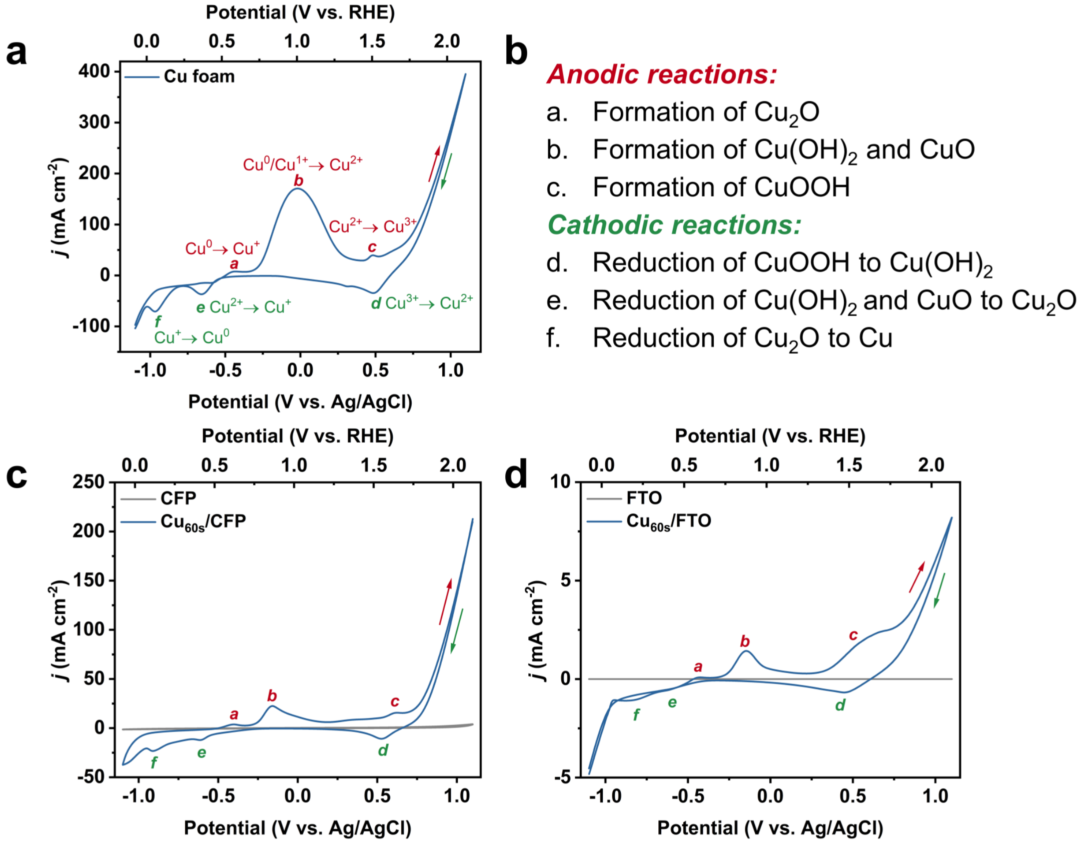

Figure S29. (a) CV curves of Cu foam in 1.0 M KOH solution. (b) Anodic and cathodic reactions of the Cu foam. CV curves of Cu_{60s}/CFP (c) and Cu_{60s}/FTO (d) in 1.0 M KOH. Scan rate: 100 mV s^{-1}.

As shown in CVs (**Figure 3a**) and in-situ Raman spectroscopy **(Figure 4b-4e**) of $NiCu_{60s}$/NF, the characteristic Cu species peaks are absent due to its small deposition amount compared with the strong Ni substrate peak. Therefore, to exclude the interference of Ni in identifying the Cu active species, Cu foam was used as the substrate.

To verify if the Cu foam is a proper substrate to identify the active Cu species, carbon fiber paper (CFP) and fluorine-doped tin oxide (FTO) were used as substrates for Cu deposition with the same deposition condition as that of $NiCu_{60s}$/NF. The corresponding samples were denoted as Cu_{60s}/CFP and Cu_{60s}/FTO.

As shown in Figure **S29a**, three anodic peaks and three cathodic peaks were identified when Cu foam was employed. The peak **a** at ~-0.4 V vs. Ag/AgCl is attributed the Cu^0 to Cu^{1+}, while the peak **b** (-0.2 V to 0 V vs. Ag/AgCl) might be assigned to two possible oxidation reactions: Cu^0 to Cu^{2+} and Cu^{1+} to Cu^{2+}. The following oxidation peak **c** at ~0.5 V vs. Ag/AgCl is associated with the conversion of Cu^{2+} to Cu^{3+}. The Cu^{3+} species can be ascribed to the formation of CuOOH, confirming CuOOH is the Cu active

species for EGOR and OER.[7, 8] The corresponding anodic and cathodic reactions are displayed in **Figure S29b**. In comparison, the CV curves of Cu_{60s}/CFP (**Figure S29c**) and Cu_{60s}/FTO (**Figure S29d**) exhibited similar redox peaks, suggesting that Cu species on different substates possess the same active species during the EGOR and OER.

2.8 In-situ Raman Section

In-situ Raman studies Cu foam

Subsequently, Cu foam was directly applied for the in-situ Raman measurement to investigate the active species of Cu during OER and EGOR.

It is worth noting that although previous works demonstrate that CuOOH is the active Cu^{3+} species for OER and biomass oxidation reaction via CV and XPS,[7-9] none of them provided direct evidence to confirm the existence of CuOOH under in-situ electrochemical conditions. This causes the assignment of CuOOH in the Raman spectroscopy is still a mystery. Thus, DFT calculation of CuOOH was conducted here to provide the necessary theoretical support.

Most previous works assigned peak-**c** to Cu^{2+} to Cu^{3+} conversion to form a CuOOH active species with formal Cu^{3+} ($3d^8$) under the alkaline condition.[7, 8] Therefore, in this work, the active Cu^{3+} species for OER and EGOR are still ascribed to CuOOH (Cu^{3+}, $3d^8$). Under OER condition (**Figure S30a**), with applied potentials between 1.32 V and 1.62 V vs. RHE, a small peak at ca. 483 cm^{-1} was observed, which is ascribed to the formation of $Cu(OH)_2$.[10, 11] With the applied potential increased over 1.62 V vs. RHE, a new peak at 570 cm^{-1}, corresponding to the formation of CuOOH[12] species, was detected. This result is supported by the DFT calculation as shown in **Figure S30c** where vibration peaks at ~500 cm^{-1} and ~550 cm^{-1} were noticed. Under the EGOR condition, besides the peak from $Cu(OH)_2$ at 491 cm^{-1}, the characteristic peak of CuOOH was observed (at ~565 cm^{-1} with an applied bias of 1.52 V vs. RHE > Cu^{2+}/Cu^{3+} redox potential of 1.50 V vs. RHE). Therefore, in-situ formed $Cu(OH)_2$ and CuOOH simultaneously served as the active species for OER and EGOR.

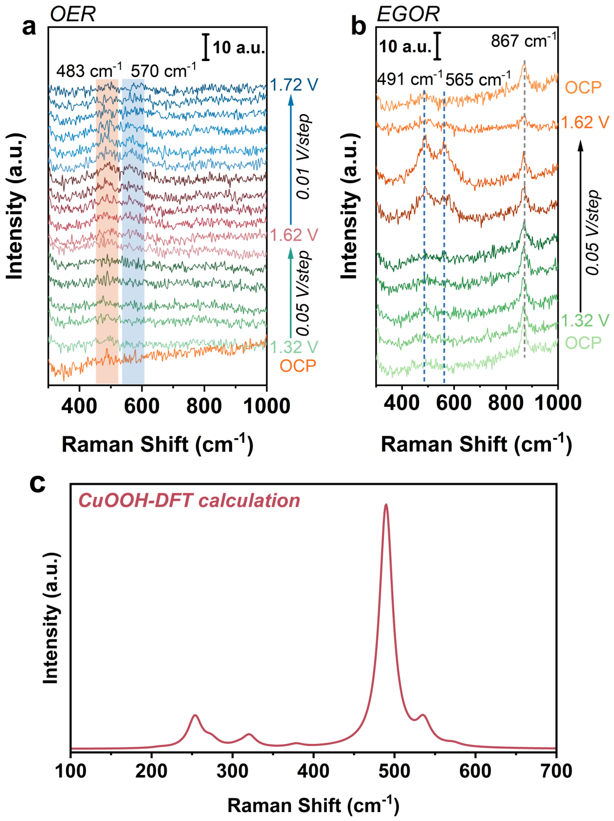

Figure S30. In-situ Raman spectra of (a) Cu foam in OER and (b) EGOR. The peak at 867 cm^{-1} is ascribed to C-C stretching vibration from EG.[13] (c) Simulated CuOOH Raman spectra by DFT calculation.

2.9 Reaction Mechanism Section

Indirect oxidation mechanism and direct oxidation mechanism

Indirect oxidation mechanism (**Scheme S1**)**:** in the indirect oxidation process, $Ni(OH)_2$ will be electrochemically oxidized to NiOOH (1), serving as a chemical oxidant and abstracting the α-hydrogen from alcohol, which is the rate-determining step (RDS) (3). Following the RDS, NiOOH will be converted back to $Ni(OH)_2$ (4). This process will be cycled by applying a bias to regenerate NiOOH from $Ni(OH)_2$. For indirect oxidation, as long as the applied potential is sufficiently positive to regenerate NiOOH, the rate of indirect alcohol oxidation is potential-independent because the RDS is a chemical step without the involvement of electricity.

$$OH^- + Ni(OH)_2 \underset{fast}{\overset{-e^-}{\rightleftharpoons}} NiOOH + H_2O \quad (1)$$

$$RCH_2OH_{sol} \rightleftharpoons RCH_2OH_{ads} \quad (2)$$

$$(RCH_2OH)_{ads} + NiOOH \xrightarrow{RDS} R\dot{C}HOH_{ads} + Ni(OH)_2 \quad (3)$$

$$R\dot{C}HOH_{ads} + 3NiOOH + H_2O \rightarrow\rightarrow\rightarrow RCOOH_{sol} + 3Ni(OH)_2 \quad (4)$$

Scheme S1. (a) Indirect oxidation mechanism for alcohol oxidation on NiOOH electrodes under alkaline condition proposed by Fleischmann.[14-16]

Direct oxidation mechanism: A few previous works reported that alcohol oxidation via the NiOOH catalyst is potential-dependent (PD) when the applied potentials are more positive than the potentials that enable indirect oxidation. This PD oxidation was referred to as direct oxidation in contrast to indirect oxidation.

As reported by Kyoung-Shin Choi's work,[16] when the dominant alcohol reaction is shifted from indirect to direct oxidation with an increased potential, an increased average Ni oxidation state is observed. For example, the average Ni valence is +2.57 at 0.45 V vs. Ag/AgCl for furfural oxidation (10 mM in pH = 13 KOH), but the average Ni valence is increased to +3.63 at 0.625 V vs. Ag/AgCl. The increased average Ni valence is attributed to the accumulation of Ni^{4+}. Therefore, the direct oxidation mechanism was proposed via hydride transfer on NiO_2 sites as shown in **Scheme S2**.

$$OH^- + Ni(OH)_2 \underset{fast}{\overset{-2e^-}{\rightleftharpoons}} NiO_2 + 2H_2O \quad (1)$$

$$OH^- + RCH_2OH \rightleftharpoons RCH_2O^- + H_2O \quad (2)$$

$$RCH_2O^-{}_{sol} \rightleftharpoons RCH_2O^-{}_{ads} \quad (3)$$

$$RCH_2O^-{}_{ads} + NiO_2 \xrightarrow[transfer]{hydride} RCHO_{sol} + Ni(OH)O^- \quad (4)$$

$$Ni(OH)O^- + HO^- \xrightarrow{2e^-} NiO_2 + H_2O \quad (5)$$

Scheme S2. Proposed PD oxidation mechanism of alcohols to aldehydes via hydride transfer.[17]

To confirm EG was indirectly oxidized by R-NiCu/NF without applied potential. Firstly, the $NiCu_{60s}$/NF catalyst was reconstructed to R-$NiCu_{60s}$/NF. Subsequently, R-$NiCu_{60s}$/NF was immersed into a 1.0 M KOH solution with the addition of 0.3 M EG. After certain reaction time, the products were identified by ^{1}H NMR as shown in **Figure S31a**.

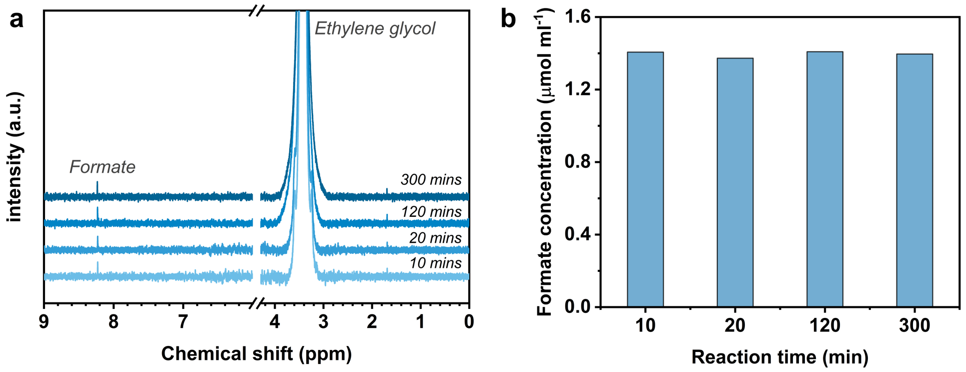

Figure S31. (a) ^{1}H NMR spectra of indirect EGOR products after different reaction times. (b) Formate concentration as a function of indirect oxidation time.

To quantitively decouple the contribution of indrect and direct oxidation, a three-step electrochemical procedure (**Figure S32**) was applied.

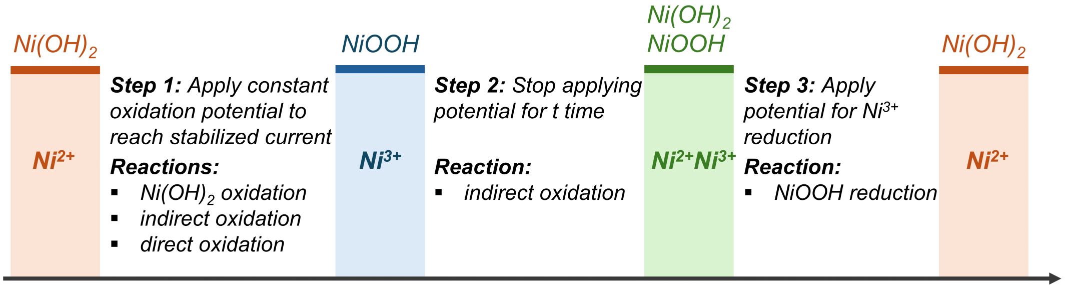

Figure S32. Schematic illustration of the 3-step electrochemical method to deconvolute direct and indirect processes.[16]

Quantitative methods description:

In the quantitative method (**Figure S32**), the first step is to apply an oxidation potential (E_1) to $Ni(OH)_2$ to oxidize it to NiOOH within a certain time to achieve a steady-state current (I_1). Herein, E_1 is less positive than the onset potential of OER to avoid any OER influence. In this step, $Ni(OH)_2$ is converted to NiOOH, and both indirect and direct reactions occur, thus, $I_1 = I_{indirect} + I_{direct}(1)$. Once the I_1 is established, the applied oxidation potential (E_1) is withdrawn and the electrolyte remains under stirring (step 2) for a t time, where only indirect oxidation will occur due to the direct oxidation is a potential-dependent reaction. In this step, NiOOH will chemically oxidize alcohol while itself will be reduced back to $Ni(OH)_2$. In the third step, a reducing potential is applied to reduce the remaining Ni^{3+} in the second step to $Ni(OH)_2$. The passed charge number (stored charge, Q) in step 3 is equivalent to the charge needed to reduce all remaining Ni^{3+} to Ni^{2+}. By repeating this 3-step process with different stirring time (t) under the open circuit condition, the disappearance of positive charge as a function of time (t) in step 2 can be obtained.

In the reported work with NiOOH as catalyst, the plot of 1/C vs t displays a linear relationship, therefore, the rate of disappearance of charge from catalyst at time t is calculated by a pseudo-second order rate law: $-\frac{dC(t)}{dt} = kC^2(t)(2)$, where the C(t) is the charge stored in the catalyst at time t and k is the 1/C vs. t plot slope.

Furthermore, the disappeared charge from the catalyst during the open circuit condition is used to oxidize alcohol, therefore, the instantaneous rate of the disappearance of charge from catalysts is equal to the rate of indirect oxidation at that time t: $-\frac{dC(t)}{dt} = r_{indirect}(t) = I_{indirect}(t)(3)$. Here, the instantaneous rate of indirect process at t = 0 s during step 2 corresponds to the steady state partial current for indirect oxidation (I_{indirect}) at the applied potential in step 1 due to the unit of rate is coulombs per second (C/s). Therefore, once I_{indirect} is determined by solving equation 2, the I_{direct} can be calculated by solving equation 1.

Indirect and direct oxidation contribution for EGOR over $NiCu_{60s}$/NF:

In this work, the developed $NiCu_{60s}$/NF displayed a FE > 90% at 1.52 V vs. RHE (0.50 V vs. Ag/AgCl. Therefore, at this potential, this method will be accurate enough to distinguish the contribution of the indirect and direct oxidation for EGOR. If the FE is low, the partial current generated from OER cannot be ignored and equation 1 will be $I_1 = I_{indirect} + I_{direct} + I_{OER}$.

Electrochemical method details:

Electrochemical experiments were conducted by using Metrohm Autolab potentiostat (software version: Nova 2.1.4). The experimental program is shown in **Figure S33a**, an oxidation potential of 0.50 V vs. Ag/AgCl was applied for 20 s to reach the steady state current. After waiting for a certain time t, a 0 V vs. Ag/AgCl was applied to reduce the remaining Ni^{3+}.

For step 3, previous work[16] holds the reduction potential for 20 s to reduce all the remaining Ni^{3+} to Ni^{2+}. However, for $NiCu_{60s}$/NF, when applying 0 V vs. Ag/AgCl, a positive current density was generated. Therefore, if the Ni^{3+} reduction to Ni^{2+} in less than 20 s, the accumulative charge number in step 3 is not the real charge number for Ni^{3+} to Ni^{2+} reduction. To address this issue, an extra command was set for step 3 (**Figure S33b**), this cutoff can help to find the accurate charge number for reducing Ni^{3+} to Ni^{2+}.

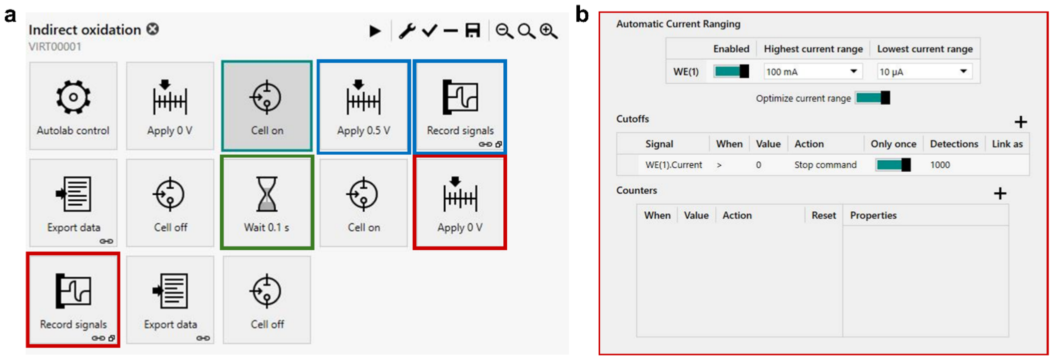

Figure S33. (a) Nova electrochemical experiment program for indirect oxidation and direct oxidation and (b) the cutoff when working electrode current > 0 is added for step 3, which is helpful for finding the accurate charge number for Ni^{3+} to Ni^{2+} reduction.

The 1/C vs. waiting time (t) was displayed in **Figure S34a**, and the current density for indirect and direct oxidation was calculated by solving equations 1 to 3. As shown in **Figure 34b**, 91.2% of the total current density originated from indirect oxidation, suggesting indirect oxidation is the dominant reaction in EGOR over $NiCu_{60s}$/NF.

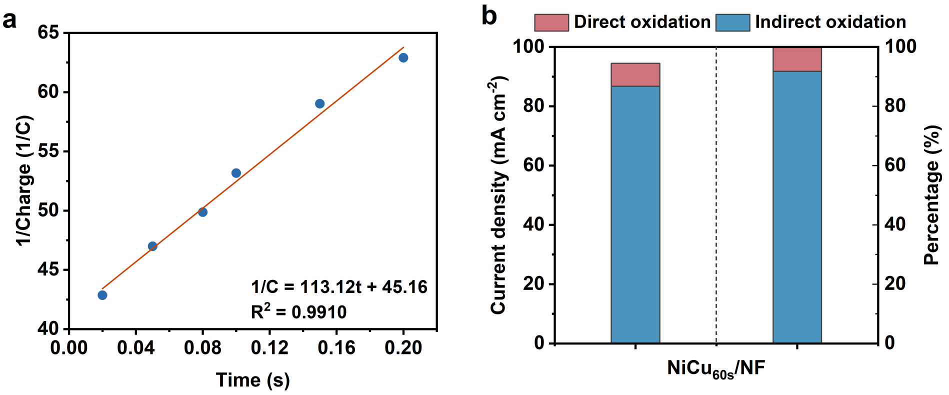

Figure S34. (a) 1/Charge vs. time plot for measuring the amount of remaining positive charge in $NiCu_{60s}$/NF after a given stirring time in 300 mM EG solution (1.0 M KOH). (b) Contribution of the indirect and direct oxidations for EGOR at 1.52 V vs. RHE.

Favorable reaction pathway

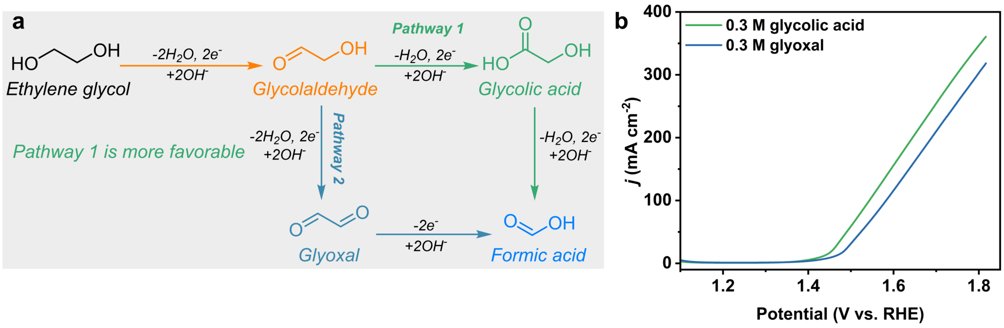

Figure S35. (a) Proposed EGOR to formic acid pathways. (b) 0.3 M glycolic acid and 0.3 M glyoxal oxidation LSV curves in 1.0 M KOH solution.

2.10 DFT calculation

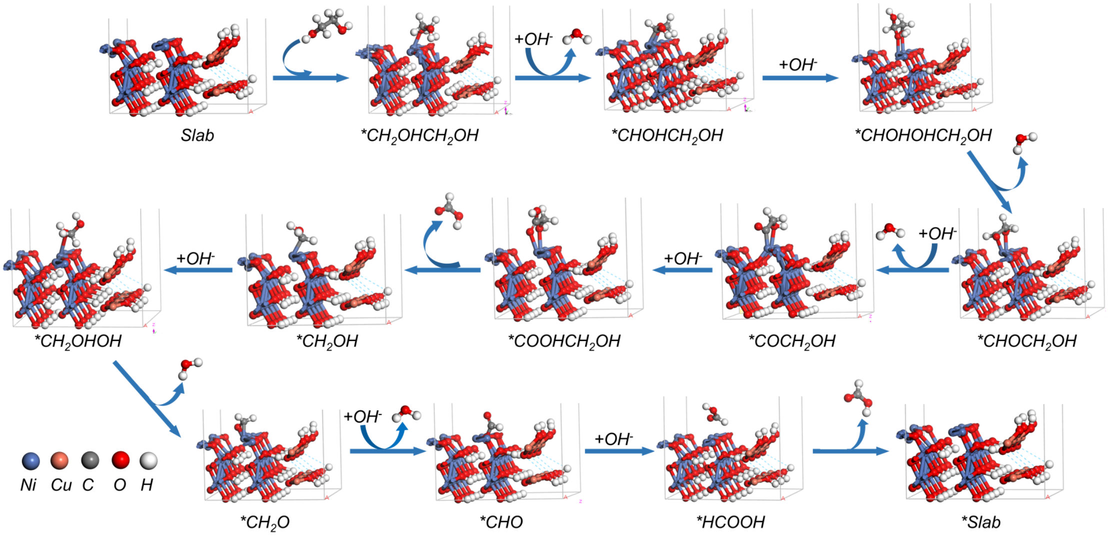

Figure S36. Optimized structures of ethylene glycol oxidation on CuOOH/NiOOH.

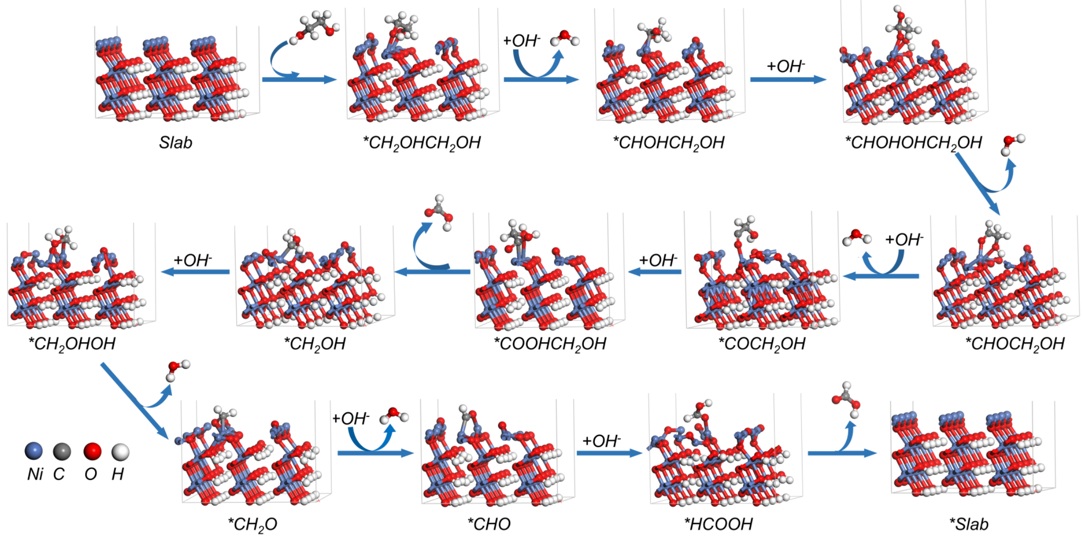

Figure S37. Optimized structures of ethylene glycol oxidation on NiOOH.

2.11 Commercial PET plastic materials upcycling

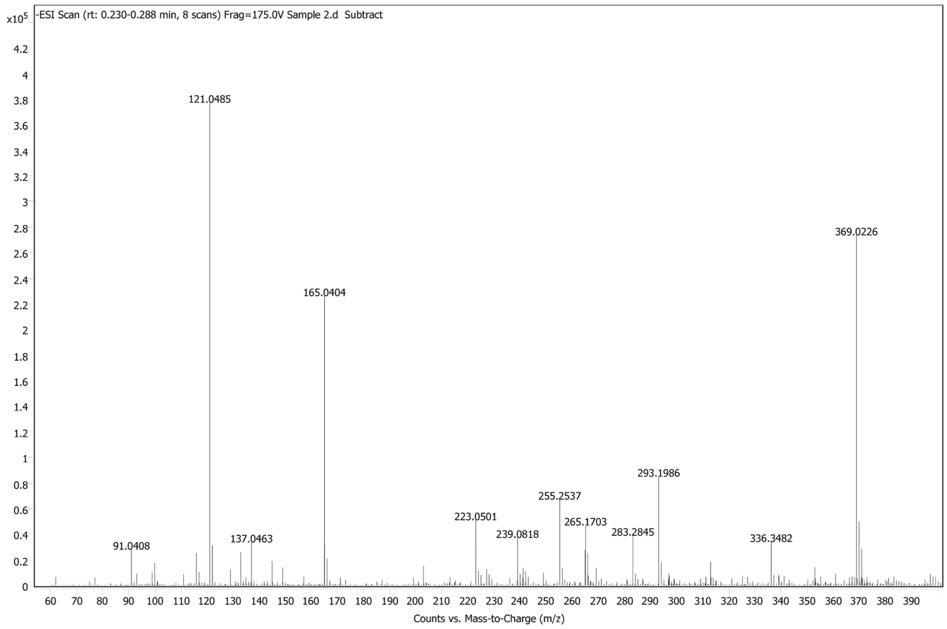

Figure S38. Liquid chromatography-mass spectroscopy (LC-MS, negative ion mode) of the PET powder hydrolysate.

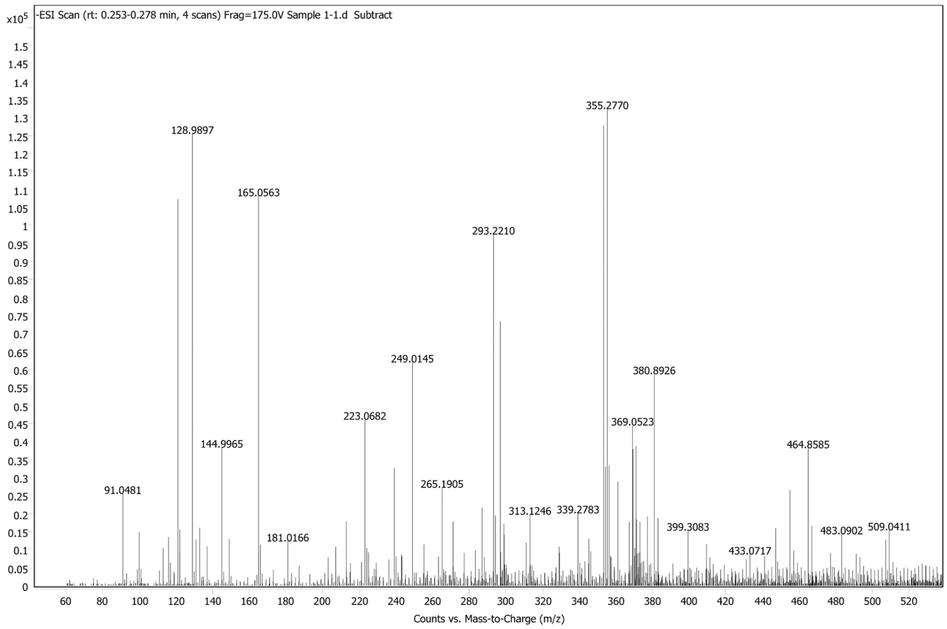

Figure S39. Liquid chromatography-mass spectroscopy (LC-MS, negative ion mode) of the commercial Aquafina PET water bottle hydrolysate

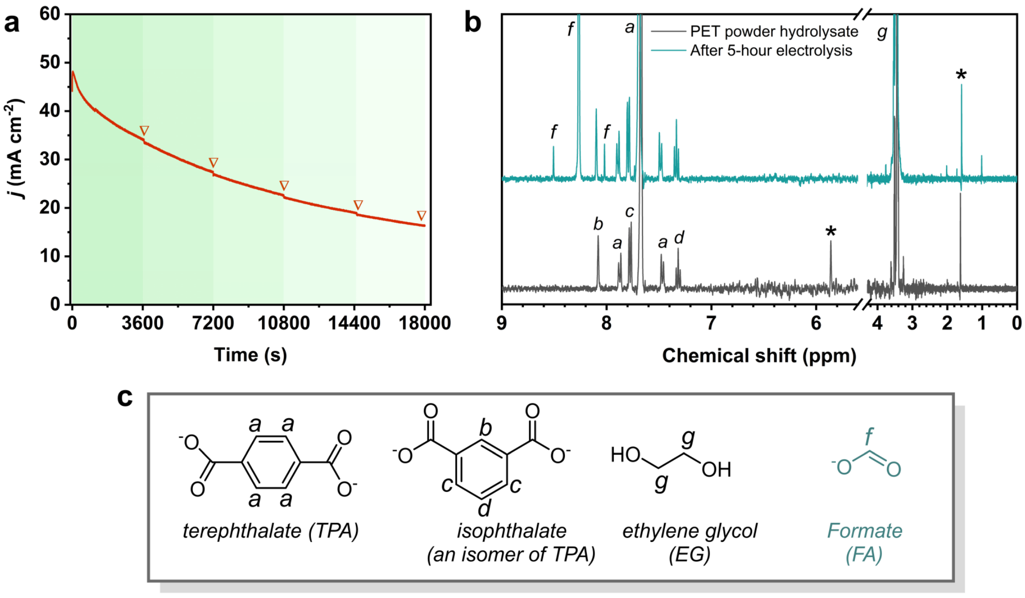

Figure S40. (a) Chronoamperometry (i-t) curve at 1.47 V vs. RHE for 5 h in PET powder hydrolysate with the $NiCu_{60s}$/NF electrode. (b) ^1H NMR of PET hydrolysate before and after 5-hour electrolysis at 1.47 V vs. RHE, (*) was also observed from control experiments. (c) Corresponding peak assignments and chemical structures.

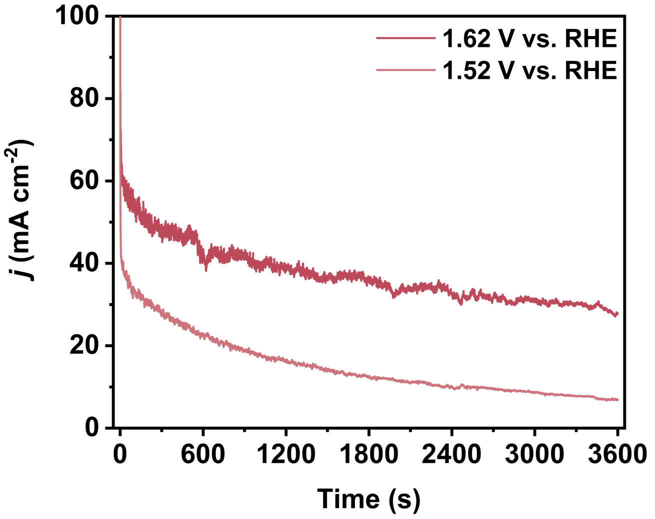

Figure S41. Chronoamperometry (i-t) curves at 1.52 V and 1.62 V vs. RHE in PET water bottle hydrolysate with the $NiCu_{60s}$/NF electrode.

3. Supplementary tables

Table S1. XPS peak binding energies and assignments of $NiCu_{60s}$/NF and R-$NiCu_{60s}$/NF.

Sample	Core level	Binding energy (eV)	Assignments
$NiCu_{60s}$/NF	Ni $2p_{3/2}$	855.3	Ni^{2+} (in $Ni(OH)_2$)[18]
	Cu $2p_{3/2}$	932.5	Cu^{+}/Cu^{0} [19]
		934.5	Cu^{2+} (in CuO)[20, 21]
	O 1s[22, 23]	529.4	M-O
		530.7	-OH
		531.9	H_2O
R-$NiCu_{60s}$/NF	Ni $2p_{3/2}$	854.5	Ni^{2+} (in NiO)[18]
		855.6	Ni^{2+} (in $Ni(OH)_2$)
	Cu $2p_{3/2}$	932.1	Cu^{+}/Cu^{0}
		933.6	Cu^{2+} (in CuO)
		935.0	Cu^{2+} (in $Cu(OH)_2$)[24]
	O 1s	528.7	M-O
		530.4	-OH
		531.7	H_2O

Table S2. Quantitative analysis of different O species obtained by curve fitting the XPS peaks.

Samples	Binding energy (eV)	Assignments	Percentage (%)
$NiCu_{60s}$/NF	529.4	M-O	3.00
	530.7	-OH	56.42
	531.9	H_2O	40.58
R-$NiCu_{60s}$/NF	528.7	M-O	19.85
	530.4	-OH	71.43
	531.7	H_2O	8.72

Table S3. Air exposure effects on identifying the oxidized Cu species.

Samples	Air exposure time (h)	Cu $2p_{3/2}$ main peak		Satellite peak area	Cu $2p_{3/2}$ peak area	CuO (%)*
		Cu^{2+} (%)	Cu^{+}/Cu^{0} (%)			
$CuNi_{60s}$/NF	3	37.82	62.18	111.7	939.5	10.63
$CuNi_{180s}$/NF	3	NA	100	18.3	3753.7	0.49
$CuNi_{60s}$/NF	45	58.80	3.64	626.8	1193.6	34.43
$CuNi_{180s}$/NF	45	45.10	33.80	516.1	2185.3	19.10

* The percent of CuO was determined by calculating the ratio of Cu $2p_{3/2}$ satellite peak area to total peak area (Cu $2p_{3/2}$ main peak + Cu $2p_{3/2}$ satellite peak).

Table S4. Quantitative analyses of surface chemical species on $NiCu_{60s}$/NF before and after 5-min Ar^+ sputtering.

**Core levels**	**Chemical species**	**$CuNi_{60s}$/NF**	
		Without Ar^+ sputtering	With Ar^+ sputtering
**Cu $2p_{3/2}$**	**Cu^{2+} (%)**	58.80	37.41
	**Cu^+/Cu^0 (%)**	3.64	46.56
	**Satellite peak area**	626.8	452.5
	**Cu $2p_{3/2}$ peak area**	1193.6	2501.8
	**CuO (%)[a]**	34.43	15.32
**Ni $2p_{3/2}$**	**NiO /$Ni(OH)_2$[b]**	0	0.24
**O 1s**	**M-O (%)**	4.54	47.61
	**-OH (%)**	45.98	50.53
	**H_2O (%)**	49.48	1.86

[a] The percent of CuO was determined by calculating the ratio of Cu $2p_{3/2}$ satellite peak area to total peak area (Cu $2p_{3/2}$ main peak + Cu $2p_{3/2}$ satellite peak).

[b] Peak area ratio of NiO to $Ni(OH)_2$ under Ni $2p_{3/2}$ main peak.

Table S5. The related EIS fitting parameters for different electrodes.

Sample	R1	R2	CPE1-T	CPE1-P
**$NiCu_{60s}$/NF**	0.7651	1.296	0.02981	0.90457
**Ni foam**	0.7708	6.477	0.00989	0.76342
**Cu foam**	0.8620	7.726	0.01341	0.82705

Table S6. A literature survey of ethylene glycol oxidation and PET electrochemical upcycling with non-noble metal electrocatalysts in the alkaline electrolyte.

Catalysts	Electrolyte	Main product and maximum of FE	Applied potential vs. RHE	Activity mA cm^{-2}@RHE	Ref
**NiCu$_{60s}$/NF**	**0.3 M EG + 1 M KOH**	**formate (95.8%)**	**1.47 V**	**100@1.45V**	**This work**
**NiCu$_{60s}$/NF**	**PET hydrolysate**	**formate (~90%)**	**1.47 V**	**100@1.45 V**	**This work**
**Ni foam**	**0.3 M EG**	**formate (74.0%), glycolate (1.79%)**	**1.47 V**	**100@1.58 V**	**This work**
**Cu foam**	**0.3 M EG**	**formate (88.9%)**	**1.47 V**	**100@1.55 V**	**This work**
$CoNi_{0.25}P$	0.3 M EG + 1 M KOH	formate (91.3 %), glycolate (~2%)	1.70 V	350@1.7 V	1[25]
CuO NW	0.1M KOH + 10 mM EG	formate (88%)	1.35 V-1.50 V	10@1.38 V	2[26]
CuO NW	0.1 M PET hydrolysate	formate (~85%)	1.40 V	10@1.38 V	
$NiCo_2O_4$/CFP	0.1 M PET hydrolysate	formate (~90%)	1.30 V-1.60 V	50@~1.43 V	3[27]
$NiSe_2$ NPs/C	1 M KOH + 1 M EG	formate (81.6%) oxalate (4.7%), glycolate (9.3%)	n.a	61.6@1.6 V	4[28]
$NiSe_2$ bundles/C	1 M KOH + 1 M EG	formate (83.5%), oxalate (4.1%), glycolate (5.6%)	n.a	81.8@1.6 V	
Branched $NiSe_2$/C	1 M KOH + 1 M EG	formate (83.4%), oxalate (5.4%), glycolate (7.4%)	n.a.	103.6@1.6 V	
FeCoNi/C	1 M KOH + 1 M EG	glycolate (~40%), oxalate (~40%)	0.4 V	~18@1.2	5[29]
FeCoNi/C	1 M KOH + 1 M EG	glycolate (36.0%), oxalate (43.5%)	1.0 V	n.a.	6[30]

n.a. is the abbreviation of “not available” from the published literature.

4. References

(1) Nørskov, J. K.; Rossmeisl, J.; Logadottir, A.; Lindqvist, L.; Kitchin, J. R.; Bligaard, T.; Jónsson, H., Origin of the Overpotential for Oxygen Reduction at a Fuel-Cell Cathode. *J. Phys. Chem. B* **2004,** *108* (46), 17886-17892.

(2) Woo, J.; Moon, B. C.; Lee, U.; Oh, H.-S.; Chae, K. H.; Jun, Y.; Min, B. K.; Lee, D. K., Collaborative Electrochemical Oxidation of the Alcohol and Aldehyde Groups of 5-Hydroxymethylfurfural by NiOOH and $Cu(OH)_2$ for Superior 2,5-Furandicarboxylic Acid Production. *ACS Catal.* **2022,** *12* (7), 4078-4091.

(3) Bale, C. W.; Bélisle, E.; Chartrand, P.; Decterov, S. A.; Eriksson, G.; Gheribi, A. E.; Hack, K.; Jung, I. H.; Kang, Y. B.; Melançon, J.; Pelton, A. D.; Petersen, S.; Robelin, C.; Sangster, J.; Spencer, P.; Van Ende, M. A., FactSage thermochemical software and databases, 2010–2016. *Calphad* **2016,** *54*, 35-53.

(4) Walters, L. N.; Huang, L.-F.; Rondinelli, J. M., First-Principles-Based Prediction of Electrochemical Oxidation and Corrosion of Copper under Multiple Environmental Factors. *J. Phys. Chem. C* **2021,** *125* (25), 14027-14038.

(5) Biesinger, M. C., Advanced analysis of copper X-ray photoelectron spectra. *Surf. Interface Anal.* **2017,** *49* (13), 1325-1334.

(6) Biesinger, M. C.; Lau, L. W. M.; Gerson, A. R.; Smart, R. S. C., Resolving surface chemical states in XPS analysis of first row transition metals, oxides and hydroxides: Sc, Ti, V, Cu and Zn. *Appl. Surf. Sci.* **2010,** *257* (3), 887-898.

(7) Pang, X.; Zhao, H.; Huang, Y.; Liu, Y.; Bai, H.; Fan, W.; Shi, W., In Situ Electrochemical Reconstitution of CF–CuO/CeO_2 for Efficient Active Species Generation. *Inorg. Chem.* **2022,** *61* (23), 8940-8954.

(8) Pang, X.; Bai, H.; Zhao, H.; Fan, W.; Shi, W., Efficient Electrocatalytic Oxidation of 5-Hydroxymethylfurfural Coupled with 4-Nitrophenol Hydrogenation in a Water System. *ACS Catal.* **2022,** *12* (2), 1545-1557.

(9) Pang, X.; Bai, H.; Huang, Y.; Zhao, H.; Zheng, G.; Fan, W., Mechanistic insights for dual-species evolution toward 5-hydroxymethylfurfural oxidation. *J. Catal.* **2023,** *417*, 22-34.

(10) Anantharaj, S.; Sugime, H.; Yamaoka, S.; Noda, S., Pushing the Limits of Rapid Anodic Growth of CuO/$Cu(OH)_2$ Nanoneedles on Cu for the Methanol Oxidation Reaction: Anodization pH Is the Game Changer. *ACS Appl. Energy Mater.* **2021,** *4* (1), 899-912.

(11) Deng, Y.; Handoko, A. D.; Du, Y.; Xi, S.; Yeo, B. S., In Situ Raman Spectroscopy of Copper and Copper Oxide Surfaces during Electrochemical Oxygen Evolution Reaction: Identification of Cu^{III} Oxides as Catalytically Active Species. *ACS Catal.* **2016,** *6* (4), 2473-2481.

(12) Ostervold, L.; Daneshpour, R.; Facchinei, M.; Tran, B.; Wetherington, M.; Alexopoulos, K.; Greenlee, L.; Janik, M. J., Identifying the Local Atomic Environment of the "Cu^{3+}" State in Alkaline Electrochemical Systems. *ACS Appl. Mater. Interfaces* **2023,** *15* (23), 27878-27892.

(13) Krishnan, K.; Krishnan, R. S., Raman and infrared spectra of ethylene glycol. *Proc. Indian Acad. Sci.* **1966,** *64* (2), 111-122.

(14) Fleischmann, M.; Korinek, K.; Pletcher, D., The oxidation of organic compounds at a nickel anode in alkaline solution. *J. Electroanal. Chem. Interfacial Electrochem.* **1971,** *31* (1), 39-49.

(15) Fleischmann, M.; Korinek, K.; Pletcher, D., The kinetics and mechanism of the oxidation of amines and alcohols at oxide-covered nickel, silver, copper, and cobalt electrodes. *J. Chem. Soc., Perkin Trans. 2* **1972,** (10), 1396-1403.

(16) Bender, M. T.; Lam, Y. C.; Hammes-Schiffer, S.; Choi, K.-S., Unraveling Two Pathways for Electrochemical Alcohol and Aldehyde Oxidation on NiOOH. *J. Am. Chem. Soc.* **2020,** *142*

(51), 21538-21547.
(17) Bender, M. T.; Warburton, R. E.; Hammes-Schiffer, S.; Choi, K.-S., Understanding Hydrogen Atom and Hydride Transfer Processes during Electrochemical Alcohol and Aldehyde Oxidation. *ACS Catal.* **2021,** *11* (24), 15110-15124.
(18) Weidler, N.; Schuch, J.; Knaus, F.; Stenner, P.; Hoch, S.; Maljusch, A.; Schäfer, R.; Kaiser, B.; Jaegermann, W., X-ray Photoelectron Spectroscopic Investigation of Plasma-Enhanced Chemical Vapor Deposited NiO_x, $NiO_x(OH)_y$, and $CoNiO_x(OH)_y$: Influence of the Chemical Composition on the Catalytic Activity for the Oxygen Evolution Reaction. *J. Phys. Chem. C* **2017,** *121* (12), 6455-6463.
(19) Dutta, A.; Rahaman, M.; Mohos, M.; Zanetti, A.; Broekmann, P., Electrochemical CO_2 Conversion Using Skeleton (Sponge) Type of Cu Catalysts. *ACS Catal.* **2017,** *7* (8), 5431-5437.
(20) Sonobe, K.; Tanabe, M.; Yamamoto, K., Enhanced Catalytic Performance of Subnano Copper Oxide Particles. *ACS Nano* **2020,** *14* (2), 1804-1810.
(21) Artiglia, L.; Sushkevich, V. L.; Palagin, D.; Knorpp, A. J.; Roy, K.; van Bokhoven, J. A., In Situ X-ray Photoelectron Spectroscopy Detects Multiple Active Sites Involved in the Selective Anaerobic Oxidation of Methane in Copper-Exchanged Zeolites. *ACS Catal.* **2019,** *9* (8), 6728-6737.
(22) Zhang, N.; Feng, X.; Rao, D.; Deng, X.; Cai, L.; Qiu, B.; Long, R.; Xiong, Y.; Lu, Y.; Chai, Y., Lattice oxygen activation enabled by high-valence metal sites for enhanced water oxidation. *Nat. Commun.* **2020,** *11* (1), 4066.
(23) Bao, F.; Kemppainen, E.; Dorbandt, I.; Xi, F.; Bors, R.; Maticiuc, N.; Wenisch, R.; Bagacki, R.; Schary, C.; Michalczik, U.; Bogdanoff, P.; Lauermann, I.; van de Krol, R.; Schlatmann, R.; Calnan, S., Host, Suppressor, and Promoter—The Roles of Ni and Fe on Oxygen Evolution Reaction Activity and Stability of NiFe Alloy Thin Films in Alkaline Media. *ACS Catal.* **2021,** *11* (16), 10537-10552.
(24) Platzman, I.; Brener, R.; Haick, H.; Tannenbaum, R., Oxidation of Polycrystalline Copper Thin Films at Ambient Conditions. *J. Phys. Chem. C* **2008,** *112* (4), 1101-1108.
(25) Zhou, H.; Ren, Y.; Li, Z.; Xu, M.; Wang, Y.; Ge, R.; Kong, X.; Zheng, L.; Duan, H., Electrocatalytic upcycling of polyethylene terephthalate to commodity chemicals and H_2 fuel. *Nat. Commun.* **2021,** *12* (1), 4679.
(26) Wang, J.; Li, X.; Zhang, T.; Chen, Y.; Wang, T.; Zhao, Y., Electro-Reforming Polyethylene Terephthalate Plastic to Co-Produce Valued Chemicals and Green Hydrogen. *J. Phys. Chem. Lett.* **2022,** *13* (2), 622-627.
(27) Wang, J.; Li, X.; Wang, M.; Zhang, T.; Chai, X.; Lu, J.; Wang, T.; Zhao, Y.; Ma, D., Electrocatalytic Valorization of Poly(ethylene terephthalate) Plastic and CO_2 for Simultaneous Production of Formic Acid. *ACS Catal.* **2022,** *12* (11), 6722-6728.
(28) Li, J.; Li, L.; Ma, X.; Han, X.; Xing, C.; Qi, X.; He, R.; Arbiol, J.; Pan, H.; Zhao, J.; Deng, J.; Zhang, Y.; Yang, Y.; Cabot, A., Selective Ethylene Glycol Oxidation to Formate on Nickel Selenide with Simultaneous Evolution of Hydrogen. *Adv. Sci.* **2023,** *10* (15), 2300841.
(29) Matsumoto, T.; Sadakiyo, M.; Ooi, M. L.; Kitano, S.; Yamamoto, T.; Matsumura, S.; Kato, K.; Takeguchi, T.; Yamauchi, M., CO_2-Free Power Generation on an Iron Group Nanoalloy Catalyst via Selective Oxidation of Ethylene Glycol to Oxalic Acid in Alkaline Media. *Sci. Rep.* **2014,** *4* (1), 5620.
(30) Matsumoto, T.; Sadakiyo, M.; Ooi, M. L.; Yamamoto, T.; Matsumura, S.; Kato, K.; Takeguchi, T.; Ozawa, N.; Kubo, M.; Yamauchi, M., Atomically mixed Fe-group nanoalloys: catalyst design for the selective electrooxidation of ethylene glycol to oxalic acid. *Phys. Chem.*

Chem. Phys. **2015,** *17* (17), 11359-11366.
